# Formulation and Characterization of Novel Ionizable and Cationic Lipid Nanoparticles for the Delivery of Splice‐Switching Oligonucleotides

**DOI:** 10.1002/adma.202419538

**Published:** 2025-03-16

**Authors:** Miina Ojansivu, Hanna M. G. Barriga, Margaret N. Holme, Stefanie Morf, James J. Doutch, Samir EL Andaloussi, Tomas Kjellman, Markus Johnsson, Justas Barauskas, Molly M. Stevens

**Affiliations:** ^1^ Department of Medical Biochemistry and Biophysics Karolinska Institute Huddinge Stockholm 171 77 Sweden; ^2^ ISIS Neutron and Muon Source Rutherford Appleton Laboratory Harwell Campus Oxfordshire OX11 0QX UK; ^3^ Division of Biomolecular and Cellular Medicine Department of Laboratory Medicine Karolinska Institute Huddinge 14152 Stockholm Sweden; ^4^ Department of Cellular Therapy and Allogeneic Stem Cell Transplantation (CAST) Karolinska University Hospital Stockholm 141 86 Sweden; ^5^ Karolinska ATMP Center Karolinska Institute Huddinge 14152 Stockholm Sweden; ^6^ Camurus AB Rydbergs torg 4 Lund 224 84 Sweden; ^7^ Department of Physiology, Anatomy and Genetics Department of Engineering Science Kavli Institute for Nanoscience Discovery University of Oxford Oxford OX1 3QU UK; ^8^ Present address: Division of Nanobiotechnology Department of Protein Science SciLifeLab, KTH Royal Institute of Technology Solna Sweden

**Keywords:** drug delivery, lipid nanoparticle, oligonucleotide, small angle scattering, stochastic optical reconstruction microscopy

## Abstract

Despite increasing knowledge about the mechanistic aspects of lipid nanoparticles (LNPs) as oligonucleotide carriers, the structure‐function relationship in LNPs has been generally overlooked. Understanding this correlation is critical in the rational design of LNPs. Here, a materials characterization approach is utilized, applying structural information from small‐angle X‐ray scattering experiments to design novel LNPs focusing on distinct lipid organizations with a minimal compositional variation. The lipid phase structures are characterized in these LNPs and their corresponding bulk lipid mixtures with small‐angle scattering techniques, and the LNP‐cell interactions in vitro with respect to cytotoxicity, hemolysis, cargo delivery, cell uptake, and lysosomal swelling. An LNP is identified that outperforms Onpattro lipid composition using lipid components and molar ratios which differ from the gold standard clinical LNPs. The base structure of these LNPs has an inverse micellar phase organization, whereas the LNPs with inverted hexagonal phases are not functional, suggesting that this phase formation may not be needed for LNP‐mediated oligonucleotide delivery. The importance of stabilizer choice for the LNP function is demonstrated and super‐resolution microscopy highlights the complexity of the delivery mechanisms, where lysosomal swelling for the majority of LNPs is observed. This study highlights the importance of advanced characterization for the rational design of LNPs to enable the study of structure‐function relationships.

## Graphical abstract

Lipid nanoparticles (LNPs) of unconventional composition and defined structures are investigated as small oligonucleotide carriers. A composition is identified that outperforms Onpattro in cargo delivery to HeLa cells. The structure of these LNPs resembles a micellar cubic phase not commonly associated with high‐performing LNPs. The results shed light on the LNP structure‐function relationship and highlight the complexity of these platforms.

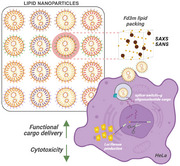

## 1 Introduction

After the success of the lipid nanoparticle (LNP) based COVID‐19 vaccine formulations delivering the mRNA for SARS‐CoV‐2 spike protein,^[^ ^1^, ^2^ ^]^ as well as the first marketed LNP drug (Onpattro) for siRNA delivery, which treats a polyneuropathy caused by hereditary transthyretin amyloidosis,^[^ ^3^ ^]^ there has been major hype around these nanocarriers for various drug delivery applications. LNPs are particularly suitable for the delivery of different types of nucleic acids, protecting them from degradation and having the potential to target the delivery to the tissue/cell type of interest. The relatively low cost, manufacturability, and the possibility to individually tailor the LNPs and cargo payload for a plethora of different medical indications have placed LNP research at the forefront of nanomedicine development. Once uptaken by cells, LNPs are trafficked through the endocytic pathway in aqueous compartments surrounded by a lipid membrane called endosomes. To effectively deliver their RNA cargo, LNPs must deliver it to the cytosol – across the endosomal membrane, also known as endosomal escape. A key issue to consider in LNP efficacy is the relationship between cell uptake and endosomal escape: there is evidence implying that these two steps do not always correlate.^[^ ^4^, ^5^, ^6^ ^]^ This adds an extra level of complexity to the LNP design. Moreover, there is still a limited understanding of the mechanistic details driving these phenomena. Currently, the fraction of LNPs among all the genomic medicines in the market or clinical trials is only ≈7%, and the majority of the clinical LNP studies are in the early phases (I‐II), indicating that there is still a long journey ahead to reach the full therapeutic potential of these drug carriers.^[^ ^7^ ^]^

One of the major obstacles to enabling higher penetration of LNP drugs to the clinic is the avoidance of liver accumulation, especially upon systemic delivery. It is possible to guide the LNPs to certain tissues/cell types, e.g., by tuning their lipid composition or introducing targeting ligands on the particle surface.^[^ ^8^, ^9^, ^10^, ^11^, ^12^ ^]^ Cheng et al. introduced the concept of selective organ targeting (SORT) where the addition of a SORT lipid to the LNP composition facilitates LNP targeting to relevant cell types in the lungs, spleen, or liver in vivo, depending on the SORT lipid and LNP charge.^[^ ^11^ ^]^ As an example of active, ligand‐based targeting, covalent antibody conjugation to the LNP surface was shown to facilitate T cell targeting and CAR T treatment of cardiac fibrosis in vivo.^[^ ^12^ ^]^ However, full control and tunability of the targeting approaches are yet to be demonstrated.

Since the chemical composition and physical arrangement of molecules in LNPs play such a big role in their performance (e.g., stability, targeting, cell uptake, endosomal escape, cytotoxicity etc.), the majority of LNP optimization has focused on compositional screening to maximize in vitro */* in vivo functional response. Several such studies have observed that small changes in the LNP components and lipid structures lead to vast differences in LNP structure and function.^[^ ^11^, ^13^, ^14^, ^15^ ^]^ Similar observations have been made for polymer and lipidoid systems, where structural changes in the individual components impacted performance and cytotoxicity.^[^ ^16^, ^17^ ^]^ However, less is known about the effect of LNP structural features (e.g., lipid packing, phase structure, and distribution) on the LNP performance in vitro and in vivo. One of the challenges with studying structure and function relationships in LNPs has been that to change LNP structure it is often necessary to change the lipid composition. This makes it almost impossible to decouple the impact of structure and lipid composition on LNP efficacy. In this publication, we address this challenge from a membrane biophysical perspective. When it comes to endosomal escape, which is possibly the biggest bottleneck in the delivery of oligonucleotides, it has been suggested that a highly curved inverted hexagonal lipid phase (H_II_), drives the LNP cargo release through membrane fusion/reorganization. This H_II_ phase is either already present in LNPs or forms as a result of the interaction between positively charged ionizable/cationic LNP lipid and negatively charged endosomal membrane in the acidic conditions of the endosome.^[^ ^5^, ^18^, ^19^, ^20^ ^]^ Moreover, highly curved and non‐bilayer lipid phases have been observed to influence other in vitro and in vivo processes including enzyme activity, membrane anchoring, and membrane fusion/fission.^[^ ^20^, ^21^, ^22^ ^]^ Recently it was shown that highly structured lipid liquid crystalline nanoparticles (H_II_, cubosome, Fd3m) were taken up better into CHO cells compared to simpler lamellar liposomes, implying a key role of the structural features of the nanoparticles.^[^ ^23^ ^]^ Also, other structural LNP features such as polyhedral or faceted shape, multilamellarity, and the presence of mRNA containing aqueous pockets on the LNP surface, induced by LNP compositional changes or by the choice of low pH buffer used for mRNA dilution during formulation, can boost mRNA transfection potency, possibly through enhanced cell uptake and endosomal escape.^[^ ^24^, ^25^ ^]^ Moreover, the distribution of the lipid components within the LNPs can impact the performance. For example, DSPC and cholesterol enrichment on the particle surface have been correlated to high transfection potency.^[^ ^6^, ^26^ ^]^ These structural features are also highly dependent on the cargo and its interactions with the lipids.^[^ ^6^ ^]^ All in all, despite these insights, the current understanding of the LNP structure‐function correlation in clinical formulations remains limited and scattered. The majority of structural characterization is more focused on highly structured LNPs, e.g., cubosomes and hexasomes. This necessitates further evaluation of structure‐function relationships for different types of LNPs to aid the rational design of novel and optimally functional delivery vehicles.

**Figure 1:**
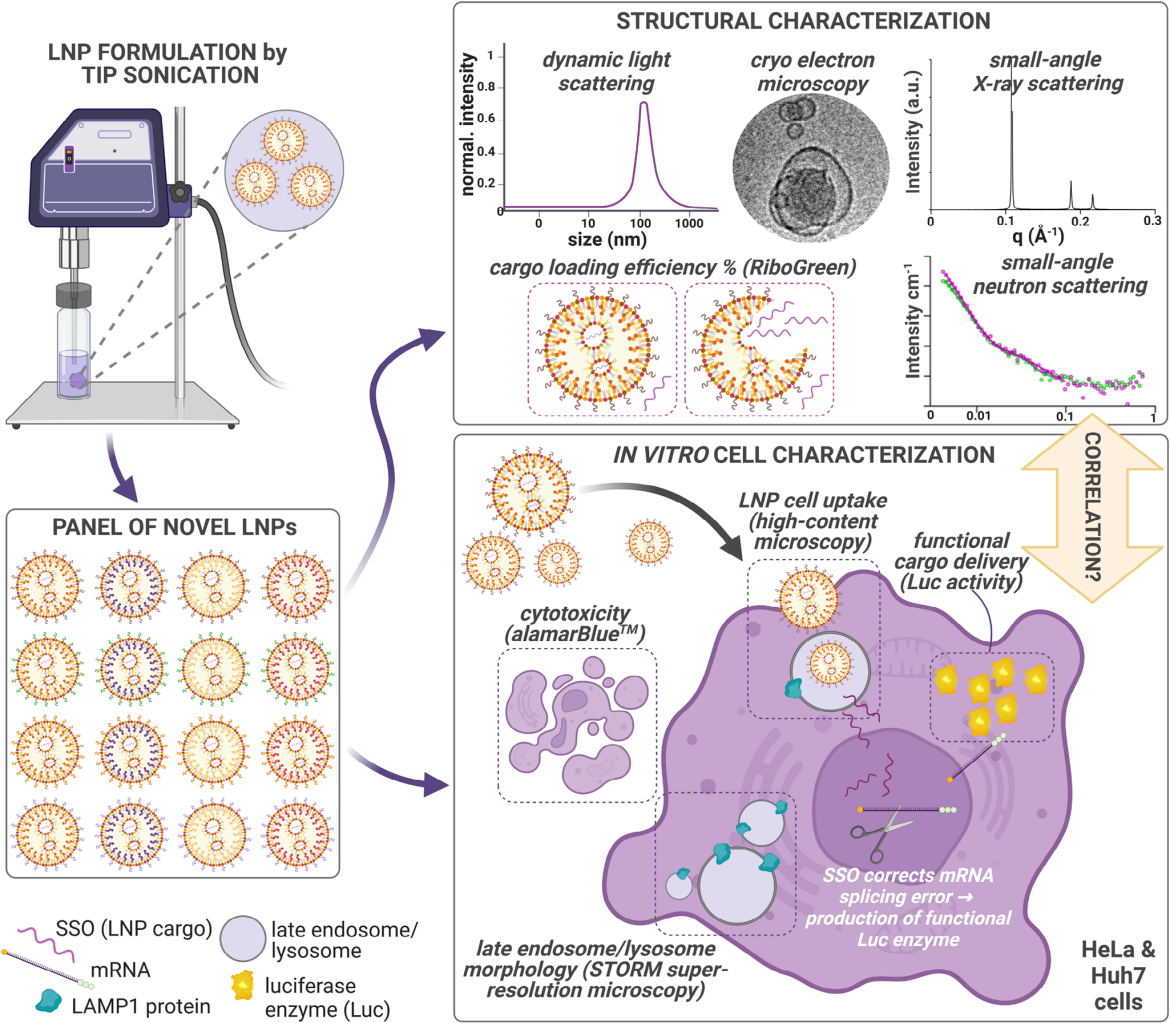
Schematic illustration of the study workflow. 16 novel LNPs, loaded with SSO, were formulated by tip sonication, followed by thorough structural characterization and evaluation of cell interactions in vitro. LNP = lipid nanoparticle, SSO = splice‐switching oligonucleotide, STORM = stochastic optical reconstruction microscopy. Figure created in BioRender (publication license acquired).

In this work, we demonstrate how a multidisciplinary approach that combines nanoscale structural characterization of novel LNPs with intracellular trafficking studies, can be used to enable the LNP research community to begin to map and unravel the complex interplay between LNP structure and successful delivery of cargo. Our approach is to work with novel lipid compositions, which do not include DPSC or cholesterol (which are commonly described as essential components in the clinical LNP formulations) and instead use two lipids that can be mixed at specific ratios to form highly ordered structures. This enables us to study structure and function in our novel formulations by changing the ratios of lipids within our LNPs but without changing the lipid components themselves. We show that LNPs with either cationic (DOTAP) or ionizable (DODAP) lipids can be formed using two novel, structurally highly ordered lipid base compositions of 1,2‐dioleoyl‐sn‐glycero‐3‐phosphocholine (DOPC): glyceryl dioleate (GDO) mixtures and a selection of different stabilizers. The use of DODAP and DOTAP, rather than other cationic/ionizable lipids, is due to their similar chemical composition. This enables us to evaluate the impact of the LNP structure on LNP performance, both via our “base lipid mixtures” and our choices of cationic/ionizable lipids whilst minimizing the chemical composition differences between formulations. The chosen compositions differ significantly from the standard compositional formula (≈50:10:38.5:1.5 mol‐% ratio of ionizable lipid, phospholipid, cholesterol, and PEG‐lipid stabilizer) used in LNPs currently in the clinic. Our model cargo, splice‐switching oligonucleotide (SSO) correcting a splicing error in engineered HeLa and Huh7 cells with non‐functional firefly Luciferase gene,^[^ ^27^ ^]^ was successfully loaded into the LNPs, as indicated by high encapsulation efficiency. Compared to the control LNPs (Onpattro composition with SSO cargo, here called MC3 LNPs), all our novel LNPs demonstrated higher cell viability, which was comparable to the non‐treated control cells. In the case of DOTAP LNPs, this is in contrast to the common observation that the cationic charge of nanoparticles is associated with higher cytotoxicity as in the context of this study, our DOTAP LNPs are not cytotoxic.^[^ ^28^, ^29^, ^30^ ^]^ Out of the 16 novel LNPs we identified a composition that outperformed the MC3 LNPs in SSO functional delivery to HeLa cells at 4 h. Whilst the majority of our LNP formulations induced an increase in the size of the intracellular structures involved in the cells degradation pathway (lysosomes), little correlation was observed between cell uptake or lysosome size and functional cargo delivery. The LNPs with the highest uptake were those containing cationic DOTAP lipid. The small‐angle X‐ray scattering (SAXS) data of bulk lipids used in the LNPs revealed that the LNPs that resulted in the highest SSO functional response were formed from a base lipid composition that adopted an inverted micellar phase structure (Fd3m). Interestingly, the LNPs formed from the hexagonal phase base composition did not result in the functional delivery of the SSO cargo. Small angle neutron scattering (SANS) data of the LNPs loaded with SSO cargo showed differences in internal LNP structures between the two base lipid compositions, as expected from the SAXS data, but little difference in surface structure (quantified via the power law). The largest differences in surface structure between LNPs were observed from LNPs formulated using different stabilizers. This correlates well with the theory that LNP stabilizers predominantly reside on the LNP surface. The outline of the current study is depicted in **Figure** **1**.

## 2 Results and Discussion

### 2.1 Rationally Selected Lipid Compositions Adopt Distinct Phase Structures and Can be Formulated into Monodisperse Nanoparticles with High Cargo Loading Efficiency

**Figure 2:**
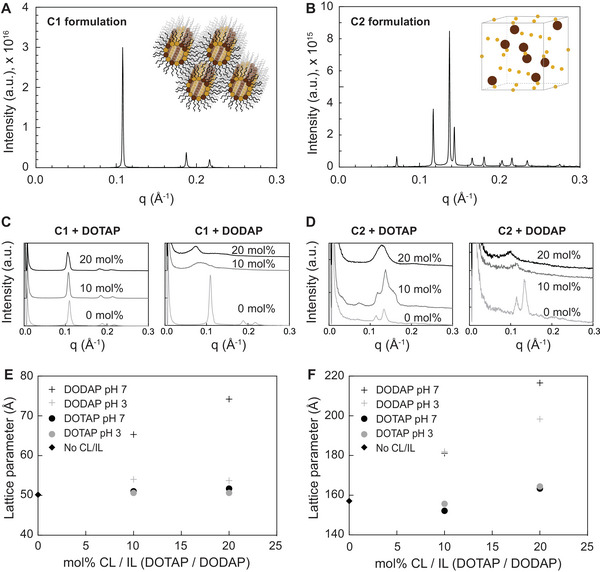
Bulk SAXS data (*N* = 1) collected at 25 °C for lipid formulations A) C1 B) C2 C) C1 + DOTAP / DODAP (0, 10, 20 mol‐%) D) C2 + DOTAP / DODAP (0, 10, 20 mol‐%). Note that the 0 mol‐% formulations plotted in C and D for DOTAP / DODAP are the same datasets, plotted twice for clarity. E) Fitted results from C1 SAXS data where error bars are of the order of the data points and reflect the error in each individual fit. F) Fitted results from C2 SAXS data where error bars are of the order of the data points and reflect the error in each individual fit.

It has been suggested that LNP structural features such as lipid packing and the H_II_ inverted hexagonal phase structure play a key role in the process of endosomal escape,^[^ ^5^, ^18^, ^19^, ^20^ ^]^ making them an attractive starting point for rational LNP design. On the other hand, it has been suggested that the siRNA encapsulated within Onpattro LNPs could reside in inverted micelle structures, and it is possible that this contributes to their high functionality when it comes to oligonucleotide delivery.^[^ ^5^, ^31^, ^32^ ^]^ One of the key challenges in this field is decoupling the effects of chemical lipid composition versus the nanoscale structure of the LNPs, as generally changes in the lipid phase structure between formulations studied at the same pH, are obtained via lipid composition changes. Inspired by these lattice structures, we investigated 2 lipid mixtures with different molar ratios of the lipids DOPC and GDO where the same two lipids could be combined in different ratios to form distinct structures. One formulation adopted a H_II_ phase (C1; **Figure** **2**A), whereas the other one had a distinct Fd3m lattice structure (C2; Figure 2B). These mixtures – C1 and C2 – served as the base lipids for our novel LNP compositions (**Table** **1**). The chemical structures of all the used lipids and stabilizers are depicted in Figure S1 (Supporting Information).

**Table 1:** Lipid nanoparticle lipid compositions in mol‐%. F127 and P80 stabilizers were added to the lipid mixtures at the point of formulation.

	Sample															
	C1								C2							
	DOTAP				DODAP				DOTAP				DODAP			
stabilizer	P80	F127	DMPE‐PEG	DSPE‐PEG	P80	F127	DMPE‐PEG	DSPE‐PEG	P80	F127	DMPE‐PEG	DSPE‐PEG	P80	F127	DMPE‐PEG	DSPE‐PEG
stabilizer mol‐%	11	0.3	2.5	2.5	11	0.3	2.5	2.5	11	0.3	2.5	2.5	11	0.3	2.5	2.5
DOTAP	20	20	20	20	–	–	–	–	20	20	20	20	–	–	–	–
DODAP	–	–	–	–	20	20	20	20	–	–	–	–	20	20	20	20
GDO	36.6	36.6	36.6	36.6	36.6	36.6	36.6	36.6	59.8	59.8	59.8	59.8	59.8	59.8	59.8	59.8
DOPC	43.4	43.4	43.4	43.4	43.4	43.4	43.4	43.4	20.2	20.2	20.2	20.2	20.2	20.2	20.2	20.2

As mentioned, obtaining structurally distinct LNPs often requires drastically changing the chemical composition, which on its own has a major effect on the functionality and therefore makes it virtually impossible to distinguish the contribution of the structure. In addition to the aforementioned base lipids, this challenge also applies to other LNP lipids, such as ionizable/cationic lipids (IL / CL). These lipids have been shown to alter the overall phase structure of the lipid mixtures,^[^ ^5^ ^]^ but also tend to clearly differ from each other chemically introducing significant compositional variation to the lipid mixtures. Here, we overcame this issue by selecting highly chemically similar CL / IL, namely DOTAP and DODAP, to be added in relatively low amounts to our base lipid compositions to enable the oligonucleotide cargo loading. DOTAP and DODAP differ only with respect to the headgroup, which in DOTAP contains a permanently positively charged ammonium group in contrast to the ionizable amine in DODAP, as well as an extra methyl (‐CH_3_) group, making the resulting LNPs compositionally highly similar and thus minimizing the effect coming from changes in the chemical composition. In other LNP studies comparing ILs MC3 and KC2 which differ only by CH_2_ in the headgroup, there is little change in the phase behavior of the LNP lipid mixture between the two lipids, even though some differences are observed in the protein expression from mRNA delivery via the different LNPs in hepatocytes in vivo.^[^ ^5^ ^]^ However, in our study, even though the differences between DOTAP and DODAP are chemically minor, they bring a distinct morphological change to the molecules and lipid mixtures. For example, DOTAP has been shown to form vesicles when hydrated in water and adopts a lamellar phase,^[^ ^33^ ^]^ whereas DODAP forms dispersions with hexagonal or Im3m morphology in water in the presence of F127.^[^ ^34^ ^]^ Thus, using DOTAP and DODAP in our formulations facilitated the evaluation of structure‐function relationships with limited changes in chemical composition in an even broader structural space.

As expected, being permanently charged, the bulk lipid mixtures containing DOTAP were not structurally pH‐responsive as indicated by the lack of pH‐induced change in the lattice parameter (swelling) detected by SAXS (Figure 2C,D; Figure S2A‐D, Supporting Information). In contrast, the bulk lipid mixtures containing ionizable DODAP demonstrated swelling in response to pH increase from 3 to 7, corresponding to the pH change from positive to neutral (DODAP pKa = 6.6^[^ ^35^ ^]^ (Bailey & Cullis, 1994)). This same effect was observed with both base compositions. In the H_II_ phase formulations, the presence of headgroup charge on the CL / IL (DOTAP, DODAP pH 3) led to smaller structures, potentially due to changes in the packing parameter of the CL / IL when the headgroup is charged. In the Fd3m phase, larger differences were observed in the lattice parameters between the CL/IL formulations irrespective of charge. In all cases, the introduction of CL / IL led to increased disorder in the bulk lipid mixtures. Swelling of IL‐containing lattices in structured LNPs has also been previously shown, although the pH‐dependent changes were observed to be highly dependent on the type of IL.^[^ ^36^ ^]^ Previous studies on bicontinuous cubic phases have shown that the introduction of charged lipids leads to a swelling of the structures – predominantly the water channels.^[^ ^37^ ^]^ In the H_II_ and Fd3m structures studied here, the mol‐% of charge introduced into the structures has less of an impact, with the lipid packing and headgroup structure also playing a significant role.

Whilst the addition of increasing amounts of DOTAP and DODAP to the C1 and C2 lipid mixtures made the structures more amorphous, some features of the H_II_ and Fd3m were still preserved (Figure 2). Generally, C1 mixtures which exhibited H_II_ phase structure retained more structure than the C2 Fd3m mixtures, and the DOTAP‐containing structures were more ordered than the DODAP ones.

**Figure 3:**
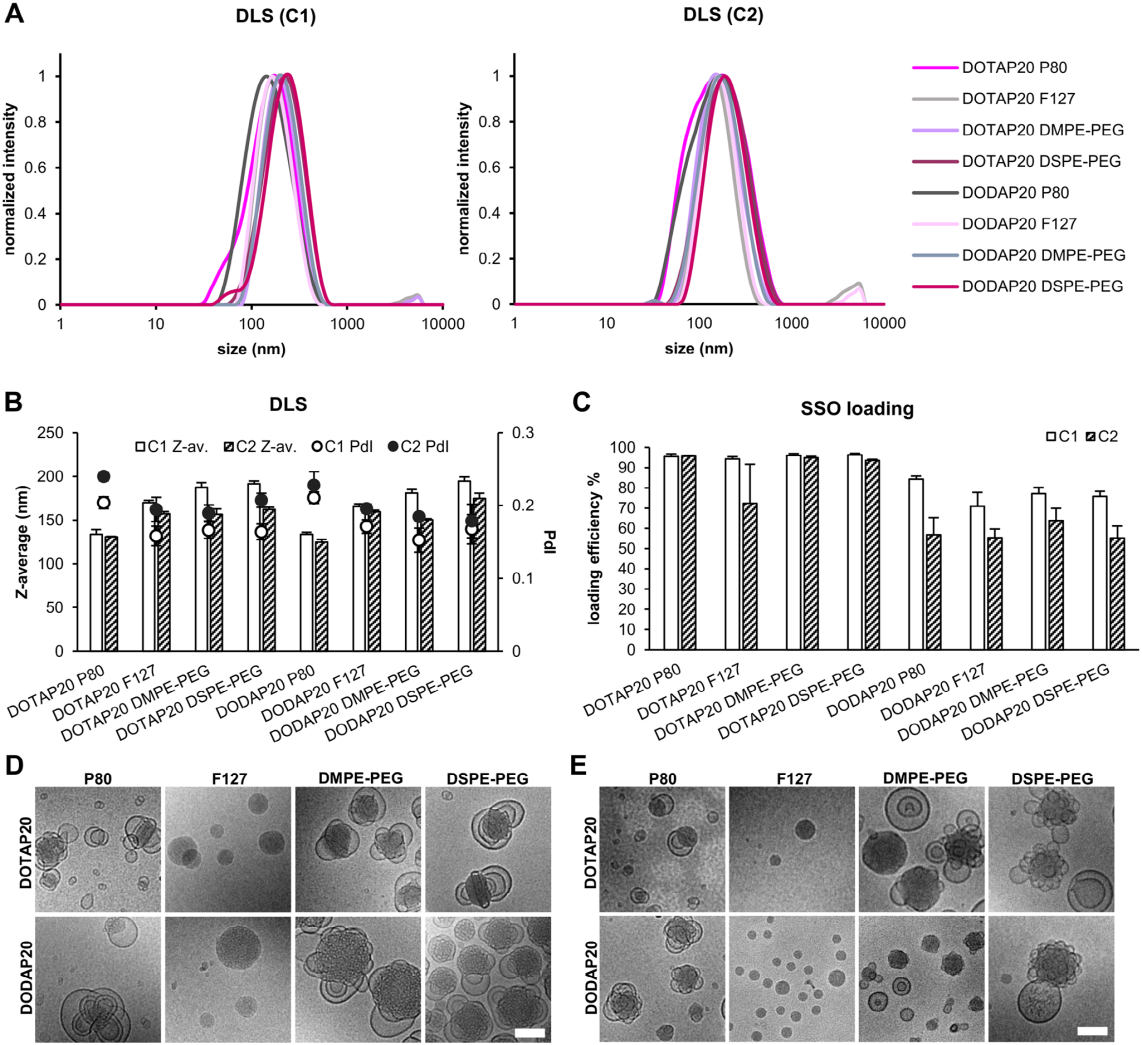
Formulation of LNPs from the novel lipid mixtures leads to high‐quality nanoparticles with low polydispersity, good cargo loading, and distinct morphologies. A) Representative DLS intensity plots for the 16 novel LNPs, measured in PBS. B) Average LNP hydrodynamic diameter (Z‐average) and polydispersity (polydispersity index, PdI) in PBS, analyzed by DLS. *N* = 3. C) SSO cargo loading efficiency % into the novel LNPs, analyzed by RiboGreen assay. *N* = 3. In B and C error bars represent standard deviation. D, E) CryoEM visualization of the morphology of novel LNP series C1 and C2 at pH 7 (PBS), respectively. Representative images are shown. Scale bars 100 nm.

To investigate the impact of these lipid structures on the delivery of SSO from LNPs, the C1 and C2 mixtures with the highest percentages of DODAP / DOTAP were formulated into nanoparticles containing the SSO cargo. The nanoparticles were stabilized with one of four different stabilizers: block copolymer Pluronic F‐127 and polysorbate 80 (P80), which are commonly used to stabilize structured LNPs,^[^ ^38^ ^]^ and PEGylated lipids DMPE and DSPE typically used in other LNP formulations.^[^ ^39^, ^40^ ^]^ The DOTAP and DODAP mol‐% in LNPs were fixed to 20 to maximize delivery but with the aim of retaining some nanoscale structure related to the base lipid compositions. As indicated by DLS, all the LNP compositions yielded good‐quality nanoparticles with PdI < 0.25 and hydrodynamic diameters ranging from 125 to 195 nm (**Figure** **3**A,B). In brief, the C1 LNPs were observed to be slightly larger and more monodisperse than the C2 ones, whereas no differences between DOTAP and DODAP particles were observed. The stabilizer used had the largest impact on the LNP size where P80 LNPs were the smallest. Our reference LNP (MC3 composition similar to that of Onpattro loaded with SSO at the same N/P ratio of 3:1) which is stabilized with 1.5 mol‐% of DMG‐c‐PEG2000 (hydrodynamic diameter 89.57nm ± 0.81 (SD), PdI 0.067 ± 0.011 (SD)) were the smallest LNPs measured. The pH was not observed to affect the size, polydispersity, or morphology of our LNP formulations (Figure S3, Supporting Information). Whilst several studies have shown that increasing the stabilizer concentration correlates negatively with LNP size^[^ ^6^, ^41^, ^42^, ^43^, ^44^ ^]^ there is less comparison of the effects of stabilizer type (e.g., block copolymer vs PEGylated lipids) on LNP size, surface structure, uptake, and functional delivery performance. Interestingly our CryoEM images show different LNP structures, dependent on both the lipid composition and the stabilizer used, although further investigation would be needed to decouple this, given that there is polydispersity in the structures and sub‐populations are also visible in the small image sampling we have of these LNPs (Figure 3D,E).

Next, we conducted a 6‐(p‐toluidino)‐2‐naphthalenesulfonyl chloride (TNS) assay to evaluate the pKa values of our LNPs since the apparent pKa is an important parameter affecting the nanoparticle performance and is known to be influenced by noncovalent interactions, environmental parameters as well as nanoparticle structural features.^[^ ^45^ ^]^ Being permanently positively charged, no apparent pKa values could be determined for the DOTAP LNPs (Figure S4A, Supporting Information). In the case of DODAP LNPs, the pKa values were observed to depend on the base lipid composition (Figure S4B, Supporting Information). In particular, pKa values were lower for the C2‐based LNPs (5.36–5.67) compared to the C1‐based ones (6.18–6.34). Moreover, F127 stabilizer resulted in an additional small decrease in the pKa. In comparison, the pKa of the control MC3 LNPs was observed to be 6.30 (Figure S4C, Supporting Information).

In addition, the encapsulation efficiency of the SSO cargo was also quantified for all the LNPs formulated (Figure 3C). The encapsulation efficiency in the DOTAP LNPs was ≈95 %, whereas for the DODAP LNPs the encapsulation efficiencies were slightly lower, ranging from ≈75 % for the C1 particles to ≈55 % for the C2 particles (Figure 3C). Semple et al. observed similar encapsulation efficiency of antisense oligodeoxynucleotides into LNPs containing 20 mol‐% DODAP, supporting our observation.^[^ ^28^ ^]^ The control MC3 LNPs had an encapsulation efficiency of 89.3 %. It has been shown that increasing the N/P ratio improves the encapsulation efficiency of both siRNA and mRNA with LNPs containing ILs.^[^ ^41^, ^42^, ^46^ ^]^ In two studies the encapsulation of siRNA and mRNA increased up to N/P of 10:1.^[^ ^41^, ^42^ ^]^ Here we used N/P 3:1 to match the Onpattro LNPs and maintain the lipid: oligonucleotide ratio during the formulation process, despite some differences in the final encapsulation efficiency. Interestingly, in the work of Ly et al., N/P ratio was not observed to have an effect on self‐amplifying RNA loading into LNPs with the tested ratios of 5, 10, and 15, suggesting that the effect of N/P ratio on cargo encapsulation might vary, especially when it comes to the type of cargo.^[^ ^47^ ^]^ Moreover, Carrasco et al.,^[^ ^48^ ^]^ showed that mRNA encapsulation efficiency did not necessarily correlate with transfection in vitro and in vivo and Whitehead et al. showed that the optimal gene silencing in vivo was achieved with 75 % siRNA loading instead of 100 %,^[^ ^49^ ^]^ which questions the necessity of maximal loading efficiency for the best performance. A key factor here is also the cargo distribution among the particles at a single‐particle level, which necessitates further evaluation in the future using methods such as single‐particle profiler.^[^ ^50^ ^]^

The visualization of SSO‐loaded LNPs with cryoEM at pH 7 revealed the presence of particles with different geometries and multiple compartments, apart from the F‐127‐containing LNPs which appeared to contain only a single lipid‐filled compartment (Figure 3D,E). No clear differences between DOTAP and DODAP or C1 and C2 LNPs were observed. The size, polydispersity, or cargo encapsulation of these LNPs used for cryoEM, which were dialyzed against PBS to reach the neutral pH, followed the same trend as the nondialyzed ones used for all the other characterizations (Figure S5, Supporting Information). The presence of so‐called “bleb” structures or aqueous pockets on the LNP surface, has been previously associated with increased mRNA transfection potency.^[^ ^24^, ^25^ ^]^ The formation of these structures has been linked to the mRNA buffer used during LNP formulation: Na‐citrate buffer, which was also used in this study, induced more such structures when compared to Na‐acetate buffer. The structural variation and distinct features revealed by cryoEM led us to conduct further structural characterization of these LNPs usingSANS as described later.

### 2.2 Novel Lipid Nanoparticle Formulation can Outperform MC3 LNPs in Certain Cell Types

**Figure 4:**
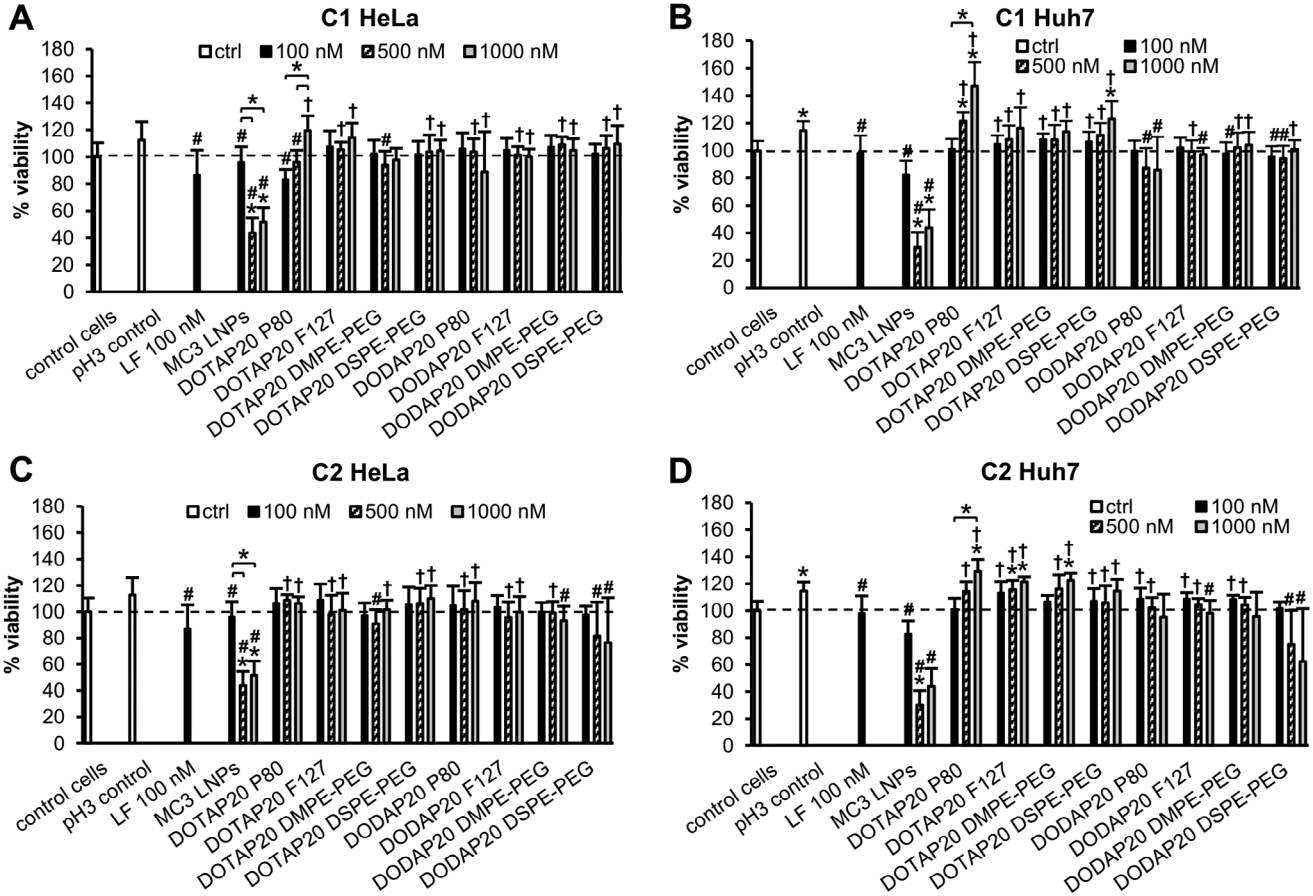
The novel LNPs demonstrate high cell viability in both HeLa and Huh7 cell models at 24 h, outperforming the MC3 LNPs (Onpattro composition). Cell viability in A,B) HeLa, and Huh7 cells treated with C1 LNPs, respectively. C,D) HeLa and Huh7 cells treated with C2 LNPs, respectively. Cell viability was evaluated with an alamarBlue assay. *N* = 3, *n* = 9 for the LNP samples, and *n* = 18 for the control cells, pH 3 control, and Lipofectamine (LF) control. * < 0.05 (compared to the control cells), # < 0.05 (compared to the pH 3 control), † < 0.05 (compared to the MC3 control), unless otherwise indicated. Kruskal‐Wallis test with Dunn's post hoc correction. Data is shown as means plus standard deviation.

Minimal cytotoxicity is a crucial prerequisite for clinically translatable LNP products.^[^ ^3^ ^]^ The cytotoxicity profile is highly dependent on both structural and compositional features of the particles. In particular, positively charged particles are commonly considered more toxic,^[^ ^29^, ^30^ ^]^ which has shifted the focus to the development of ILs, which are neutral in neutral pH and therefore overcome the charge‐related problems associated with permanently positive CLs.^[^ ^28^ ^]^ In contrast to these observations, all our novel LNPs, including the ones with cationic lipid DOTAP, showed no signs of cytotoxicity after 24 h cell treatment even with the highest doses (**Figure** **4**). It is possible that the relatively low mol‐% of DOTAP used in these particles (20 mol‐%) rendered them less toxic since the charge‐related toxicity correlates with charge density.^[^ ^51^ ^]^ Importantly, MC3 LNPs, despite being neutral at cell culture conditions, significantly decreased the cell viability at 24 h with medium and high doses, implying that these particles are less optimal in this in vitro setup compared to our novel LNPs. Moreover, this also suggests that charge is not the only factor determining the toxicity. After 4 h treatment, the cell viability was high in all samples (Figure S6, Supporting Information). Moreover, no differences between the cell lines were observed with respect to the cell viability in response to the LNP treatment.

In addition to in vitro cytotoxicity measurement, we also conducted a hemolysis assay to better understand the in vivo compatibility of our LNPs. As demonstrated in Figure S7 (Supporting Information), apart from the F127‐containing LNPs none of the studied particles induced lysis of human erythrocytes at physiological pH (7.4). Interestingly, the presence of F127, in particular in combination with the C2 base lipids, lead to more than 50 % hemolysis with the highest tested doses. Barauskas et al. have previously observed that F127 alone is not hemolytic^[^ ^52^ ^]^ but in the work of Johnsson et al., the addition of F127 to pre‐formed phosphatidylcholine vesicles/liposomes encapsulating a membrane permeability marker caused massive leakage of this marker,^[^ ^53^ ^]^ suggesting that F127 can affect the membrane permeability. In any case, no hemolysis was detected with the LNPs performing the best in cargo delivery as described below.

**Figure 5:**
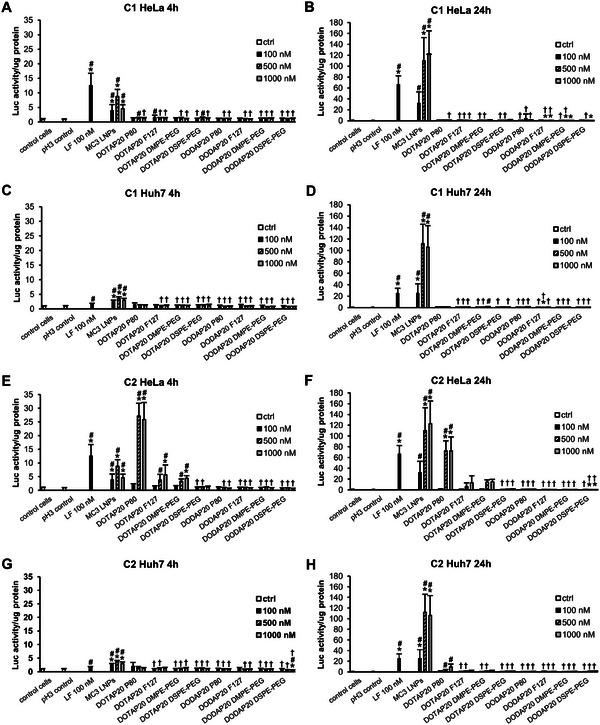
C2 LNPs containing DOTAP and P80 stabilizer outperform MC3 LNPs (Onpattro composition) in functional SSO delivery into HeLa cells at 4 h, whereas none of the DODAP LNPs or the C1 LNPs can deliver the cargo. A,B) Delivery of C1 LNPs into HeLa cells at 4 and 24 h, respectively. C,D) Delivery of C1 LNPs into Huh7 cells at 4 and 24 h, respectively. E,F) Delivery of C2 LNPs into HeLa cells at 4 and 24 h, respectively. G,H) Delivery of C2 LNPs into Huh7 cells at 4 h and 24 h, respectively. LF = Lipofectamine. *N* = 3, *n* = 9 for the LNP samples, and *n* = 18 for the control cells, pH 3 control, and Lipofectamine (LF) control. * < 0.05 (compared to the control cells), # < 0.05 (compared to the pH3 control), † < 0.05 (compared to the MC3 control), unless otherwise indicated. Kruskal‐Wallis test with Dunn's post hoc correction. Data is shown as means plus standard deviation.

To successfully deliver the cargo to the cells, the LNPs first need to reach the cell surface, enter the cell, which normally occurs through endocytosis,^[^ ^54^, ^55^ ^]^ and release the cargo from the endosomal compartments. In the case of some cargoes, such as the SSO used in this project, they even need to reach the nucleus. To study the functional cargo delivery, we used a model system of HeLa and Huh7 cells engineered to express a non‐functional firefly Luciferase (Luc) gene with a splicing error.^[^ ^27^ ^]^ This error can be corrected with the successful delivery of an SSO, leading to easily measurable Luc enzyme activity readout in the cell lysates. Dysregulation of mRNA splicing is the cause of many hereditary diseases and is also associated with cancers, making SSOs highly relevant therapeutic molecules.^[^ ^56^, ^57^ ^]^ Here, Luc activity was measured after 4 and 24 h LNP treatment (**Figure** **5**). As expected, MC3 LNPs efficiently delivered the SSO to both cell types with Luc activity increasing from 4 to 24 h. The C1 series of LNPs, irrespective of the CL / IL or the cell type, showed poor SSO delivery capacity (Figure 5A–D). Moreover, Huh7 cells could not be transfected with the C2 series LNPs either (Figure 5G,H). However, in the case of HeLa cells, C2 LNPs with DOTAP and P80 stabilizer turned out to outperform the MC3 LNPs in SSO delivery at the early time point and sustain comparable levels at the later time point (Figure 5E,F). Minor dose‐dependent functional delivery, especially at the 4 h time point, was also observed with the C2 DOTAP LNPs with F‐127 and DMPE‐PEG2000 stabilizers, whereas the rest of the C2 particles were completely unable to facilitate the cargo delivery.

Since it has been suggested that a highly curved inverted hexagonal lipid phase (H_II_) drives the LNP cargo release from endosomes through membrane fusion / reorganization,^[^ ^5^, ^18^, ^19^, ^20^ ^]^ we hypothesized that the LNPs with C1 base composition, which has this phase structure, would be efficient in functional cargo delivery. However, we observed the opposite, i.e., the only functional LNPs had the C2 base, which is organized as an inverse discontinuous micellar cubic phase (Fd3m). Yu et al. reported that in a macrophage in vitro model, the H_II_ phase LNPs were inferior to cubic nanoparticles in transfecting the cells, though in this work the effect of nanoparticle composition cannot be completely ruled out.^[^ ^58^ ^]^ The same team also demonstrated that the phase structure of LNPs formulated primarily from the SARS‐CoV‐2 vaccine lipids SM‐102 and ALC‐0315 and a stabilizer were both able to form bicontinuous cubic phases in acidic conditions with SM‐102 exhibiting this at a pH corresponding to endosomal acidification (pH 5) and ALC‐0315 exhibiting this at pH 3.^[^ ^36^ ^]^ These observations combined with our own results challenge the general view of inverted hexagonal lipid phases as a necessary lipid organization for LNP cargo to escape from the endosomes. In most cases, the ability of LNP lipids to form highly curved lipid phases appears to contribute to increased LNP performance but is not confined to a single phase morphology (i.e., inverted H_II_) as previously suggested.

MC3 ionizable lipid and Onpattro LNPs were originally optimized for liver cell transfection.^[^ ^31^ ^]^ Therefore, the good transfection capacity of the Huh7 cells, a hepatocyte‐derived carcinoma cell line, was expected. Our LNPs significantly differ both compositionally and structurally from the Onpattro LNPs, which possibly makes them less susceptible to liver cell delivery, potentially through differences in protein corona composition (e.g., ApoE binding) and dynamics. The poor transfection of liver cells with our LNPs might be beneficial for non‐liver targeting in vivo, though this necessitates further evaluation.

The size of LNPs has also been shown to affect LNP performance.^[^ ^18^, ^42^ ^]^ Interestingly, it has been observed that LNPs resembling the Onpattro composition have the best antisense oligonucleotide (ASO) delivery efficacy in vitro in the size range of 100–155 nm, where the LNPs have the lowest disordered / H_II_ ratio in their core, i.e., the highest extent of inverted hexagonal phase in the core corresponds to best performance.^[^ ^18^, ^44^ ^]^ These results were independent of the PEG‐lipid molar ratio, which did not have a significant bearing on the LNP delivery of ASO. Our LNPs with P80 stabilizer fall size‐wise into this range (Z‐average ≈130 nm), which might be one factor explaining their good performance, although the compositional base is different. On the other hand, there is also evidence that LNP sizes < 100 nm are ideal for the performance,^[^ ^42^ ^]^ suggesting that the effect of size might be highly dependent on the LNP system and cell model.

All in all, comparing the data to the existing literature is challenging due to highly varying experimental parameters (e.g., LNP composition, cargo, formulation method, post‐processing, buffers, and cell / animal models). Moreover, it is often hard or impossible to adjust only the parameter of interest in these highly dynamic systems. This complexity is demonstrated on a small scale in the work of Palchetti et al., which showed that in the panel of 16 different LNPs, none of the studied physicochemical characteristics (size, aggregation, charge, protein corona fingerprint) alone was exclusively able to account for interactions with HeLa cells.^[^ ^59^ ^]^

### 2.3 Structural Analysis with Small Angle Neutron Scattering (SANS) Enables Mechanistic Insights into LNP Performance

**Figure 6:**
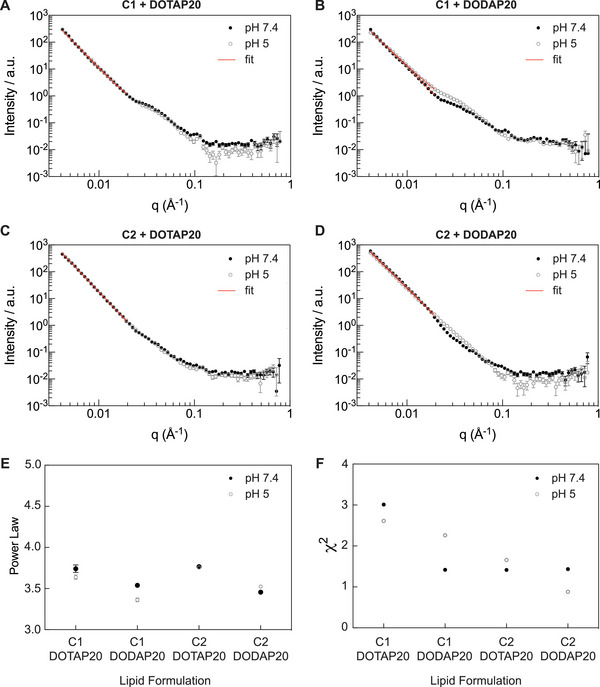
Small angle neutron scattering (SANS) data of LNP samples formulated with SSO and stabilized with P80 with lipid compositions A) C1 + DOTAP 20. B) C1 + DODAP 20. C) C2 + DOTAP 20. D) C2 + DODAP 20 measured at 37 °C in either pH 7.4 or pH 5. For A‐D. Data points are raw data with the power law fit over a truncated q range overlayed as a straight line (red). For E,F) each individual data point represents *N* = 1 with the error bars showing the error in the fit.

Coupling membrane biophysical properties of the investigated LNPs to their activity in vitro provides the opportunity to both provide structural explanations for why some formulations are more active than others and to inform the design of wider libraries of biologically active LNPs. To this end, small angle neutron scattering (SANS) was next performed on selected formulations, to understand whether any of the LNP structural properties could be related to the increased performance measured (**Figure** **6**E,F). Based on previous studies emphasizing the impact of lipid composition and internal LNP structure, the first permutation of structural analysis related to the different “base lipid” formulations (C1, C2) with a fixed SSO loading ratio and stabilizer content (P80). A qualitative assessment of the data (Figure 6A–D) shows slopes with similar gradients between 0.00416 < q < 0.02 Å^−1^ in all of the samples, with key differences between the lipid compositions observed in the region 0.02 < q < 0.2 Å^−1^. From the SAXS data of the bulk lipid mixtures, we expect the internal structure of the C1 formulations to stem from an inverted hexagonal phase (H_II_) which is supported by the small peak observed at ≈0.1 Å^−1^. The broad peak observed in the region 0.02 < q < 0.1 has previously been attributed to a bicontinuous cubic phase,^[^ ^60^ ^]^ however in this case there is no evidence of this in the SAXS data, and the invariance of the broad peak to temperature implies that this is not the case for these samples and is related to a temperature invariant structure of the order of 14 – 21 nm (assuming a peak center of 0.02–0.03 Å^−1^, where structure size = 2𝜋/q). Another alternative explanation is therefore that this broad peak is the result of a co‐population of unilamellar vesicles (as seen in the CryoEM images, Figure 3B) however further investigation would be needed to confirm these hypotheses. According to the SAXS data, C2 formulations stem from a disordered micellar cubic phase (Fd3m) with the most intense diffraction peak situated ≈0.14 Å^−1^. This peak is weakly present in the C2 DOTAP20 sample but not resolvable in the C2 DODAP20 sample which corresponds well with the SAXS data and implies a higher disorder in the C2 DODAP20 sample. As expected, upon diluting the samples into pH 5 buffer to represent the late endosomal pH^[^ ^61^ ^]^ structural changes were observed in the samples containing DODAP lipid (pKa by TNS = 5.62)^[^ ^48^ ^]^ and were observed to a much lesser extent in the samples containing DOTAP lipid (cationic).

After the qualitative assessment, the fitting of the SANS data was performed using SasView. Due to the polydispersity apparent in the samples from the CryoEM images, a shape‐independent fitting approach (power law) was adopted. The sizes of the LNPs meant that the Guinier region was not captured over this q range (see methods for further details), however, power law fits were used to analyze the Porod region, enabling a comparative assessment of the surface roughness of the LNPs (Figure 6E,F). In the samples measured and analyzed (*N* = 1), the power law for the DOTAP samples is higher than that for the DODAP samples implying a smoother LNP surface for the DOTAP samples.^[^ ^62^, ^63^ ^]^ One hypothesis is that the bulkier headgroup of the DOTAP lipid leads to a smoother surface compared to the smaller DODAP headgroup. This implies that even with the presence of PEG on the LNP surface (which sequesters solvent and is therefore likely to have low contrast compared to the solvent), the DOTAP / DODAP lipids are still able to impact the LNP surface properties. However, it should be noted that as these samples are *N* = 1, and the differences are small, further statistics would be required to confirm this.

**Figure 7:**
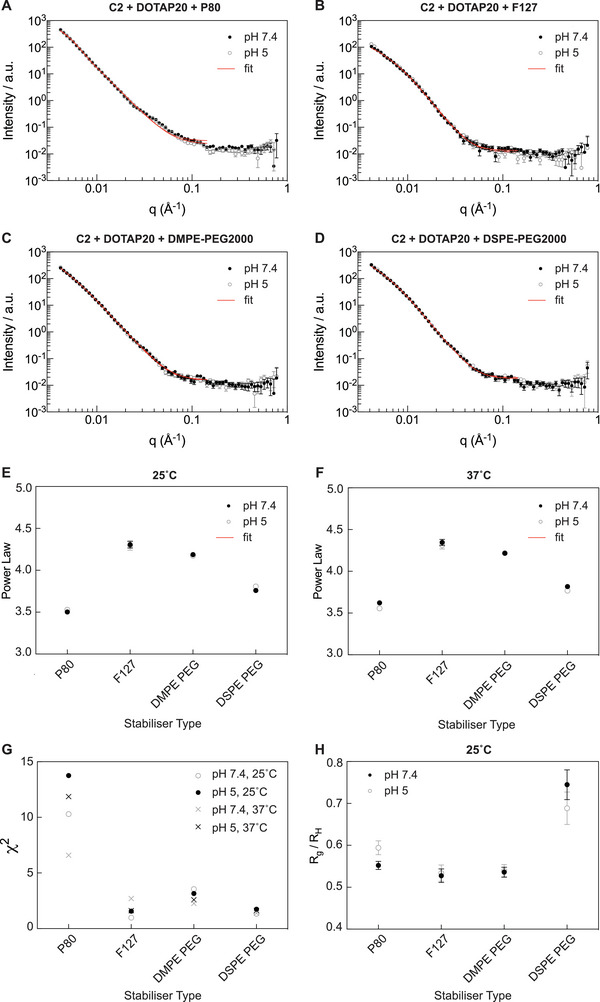
Small angle neutron scattering (SANS) data of LNP samples formulated with SSO and lipid composition C2 DOTAP 20 and stabilized with either A) P80. B) F127. C) DMPE‐PEG2000. D) DSPE‐PEG2000 and measured at 25, 37 °C in either pH 7.4 or pH 5. For A–D. Data points are raw data with the unified model fit over a truncated q range overlaid as a red line. For E–H) each individual data point represents an individual fit where *N* = 1 and the error bars represent the error in the fit.

After analyzing the impact of lipid composition on LNP structure using SANS, we further probed the lead formulation (C2 DOTAP 20) to study the impact of the stabilizing agent used. This was particularly interesting as while the C2 DOTAP 20 lipid formulation was the only active formulation, the stabilizer used to formulate the LNPs also impacted the cellular performance (LNPs prepared with P80 were significantly more active). From a qualitative assessment of the SANS data, there were observable differences between the SANS curves despite the lipid composition being conserved across samples and only the stabilizer varied (0.3–11 mol‐%), which is proportionally a minimal component of the LNPs. These differences appeared to relate not only to the LNP size but also to the overall structure. This agrees with the CryoEM data in Figure 3E, although the populations of LNPs observed there represent only a small fraction of the LNPs, whereas the SANS is an average of the scattering of the whole sample in the neutron beam. As expected, with the CL DOTAP, there was no significant pH dependence of the sample structure between pH 5 and pH 7.4. For this LNP sample set, the Guinier region was partially captured and therefore a Unified Model fit was applied over a wider q range than for the LNPs with different lipid compositions. Results from these fits, including the differences at 25 and 37 °C and the Chi^2^ from the fits are displayed in **Figure** **7**E–G. The individual results for the R_g_ obtained from SANS fitting and R_H_ from DLS and the background measurements performed on the stabilizers in solution are included in Figure S8 (Supporting Information). At 25 °C we obtained corresponding DLS data for the same LNPs and therefore these are displayed in Figure 7H where the ratio of R_g_ (radius of gyration from the neutron fitting) and R_H_ (hydrodynamic radius from the DLS Z intensity averaged data) are displayed. The difference in the R_g_ / R_H_ ratio obtained for the DSPE‐PEG2000 containing LNP compared to the other LNPs could imply a density difference, however further investigation would be needed. The power law (Porod exponent) fits revealed greater differences in the surface roughness of the LNPs between different stabilizers when compared to different lipid compositions. Comparing these different stabilizers is challenging as they are structurally different and span molecular weights from 1.2 k Da up to 79 k Da. We also did not prepare all LNPs with equal amounts of stabilizers but instead based our mol‐% choices on screening experiments and literature precedence. If we look solely at the mol‐% of stabilizer added to our LNP formulations, the surface roughness appears to correlate with the mol‐% of stabilizer added, i.e., surface roughness is in the order (roughest to smoothest) P80 : DSPE / DMPE‐PEG2000 : F127 which have a mol‐% of 11 : 2.5 : 0.3. The interactions of these stabilizers with lipid membranes have been studied extensively and it is known that they can intercalate into the membrane.^[^ ^53^ ^]^ Based on solely studying the relative mol‐% of the stabilizers used in these experiments, it is therefore reasonable to assume that the P80 would be significantly more crowded on the LNP surface and may adopt a more extended chain conformation whereas PEG‐lipid stabilizers with smaller mol‐% may have more compact “mushroom” conformations. Bulk SAXS measurements of the lipid mixtures with stabilizer co‐dissolved (Figure S9, Supporting Information) show the most disorder in the P80 and DMPE‐PEG2000 containing samples, however further investigation is needed. Interestingly, whilst the scattering contrast of the stabilizers is expected to be low, there are still measurable differences between the different bulk samples and LNPs showing the impacts of the stabilizers on the systems.

### 2.4 Lipid Nanoparticle Uptake and LNP‐Induced Lysosome Swelling Have Minor Correlation with LNP Performance

**Figure 8:**
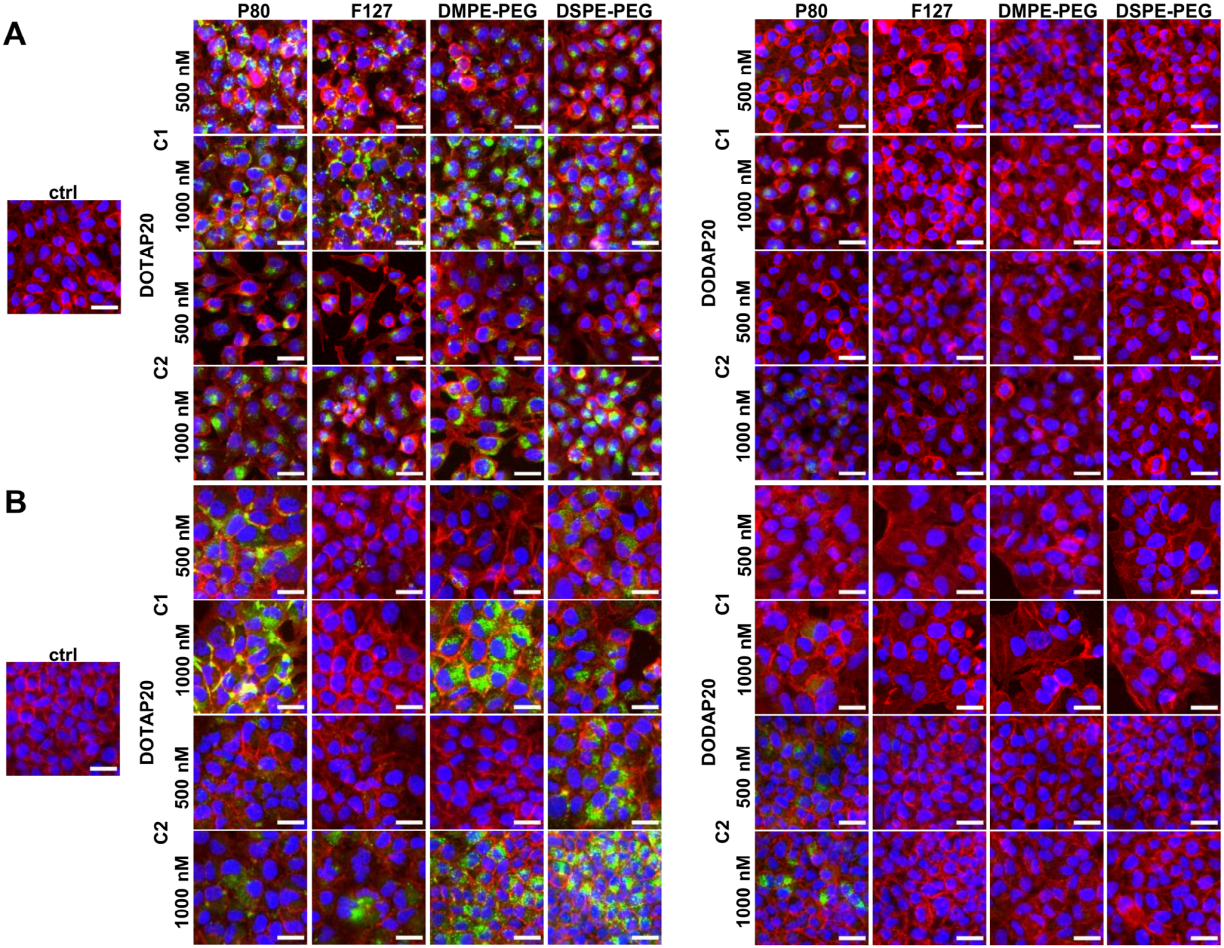
DOTAP 20 LNPs in both C1 and C2 series are generally well uptaken into both HeLa and Huh7 cells, whereas DODAP 20 LNPs do not enter the cells. A,B) Representative images of LNP uptake into HeLa and Huh7 cells at 24 h time points, respectively. DOTAP 20 LNPs on the left side and DODAP 20 on the right side. Rhodamine B‐labelled LNPs = green, actin cytoskeleton labeled with phalloidin Alexa Fluor 647 = red, nuclei labeled with DAPI = blue. Scale bars 30 µm.

Since we saw major differences in performance between the LNPs, we decided to evaluate the cell uptake to elucidate the issues related to functional delivery. Interestingly, DOTAP LNPs, irrespective of the base composition, stabilizer, or cell type, entered the cells as seen from the microscopy images of cells treated with Rhodamine B‐labelled LNPs for 24 h (**Figure** **8**). In contrast to this, all the DODAP‐containing LNPs were poorly internalized, which provides an explanation for their inability to perform functional cargo delivery. As discussed before, LNP charge can affect the cell uptake and functional cargo delivery through protein corona composition, which might be a cause of the differing cell uptake behavior between DOTAP and DODAP LNPs, however, the surface structure differences may also contribute to protein corona formation. Interestingly, we did not observe any clear differences between HeLa and Huh7 cell uptake, and the uptake at an earlier 4 h time point followed the same trend as the 24 h time point but with somewhat less internalized LNPs (Figure S10, Supporting Information).

Despite the good uptake of all the DOTAP particles, only C2 DOTAP LNPs with P80 stabilizer showed high cargo delivery efficiency. DSPE‐PEG2000 and other PEGylated lipids with long acyl chains (C18) have been shown not to shed from the LNP surface when in contact with biological fluids, hampering both the LNP uptake and functional cargo delivery.^[^ ^44^, ^64^, ^65^, ^66^ ^]^ In contrast to this, in our lipid systems the DSPE‐PEG2000 seems to negatively affect only the endosomal escape but not the LNP cell uptake. In general, a lack of correlation between cell uptake and functional cargo delivery has been also reported previously,^[^ ^4^, ^5^, ^6^ ^]^ highlighting the importance of considering the cell uptake and endosomal escape as two separate obstacles, needing separate solutions to overcome and hence representing a formidable challenge in the delivery of oligonucleotides.

Since the cell uptake patterns could not completely explain the differences in the performance of the different LNPs, we decided to dig deeper and evaluate the effect of LNP treatment on the LAMP1‐positive endolysosomal structures. Since endocytosis is assumed to be the prevalent LNP cell entry route,^[^ ^54^, ^55^ ^]^ the particles most likely end up in the endosomal compartments upon successful cell uptake. Late endosomes / lysosomes are often smaller than the ≈200 nm diffraction limit of light, meaning that their visualization requires fluorescence microscopy techniques to break the diffraction barrier. In this study we used a stochastic optical reconstruction microscopy (STORM) super‐resolution imaging approach,^[^ ^67^ ^]^ combined with a deep learning algorithm for the lysosome size quantification.^[^ ^68^, ^69^, ^70^ ^]^ DODAP‐containing LNPs were omitted from this analysis due to their low uptake levels.

**Figure 9:**
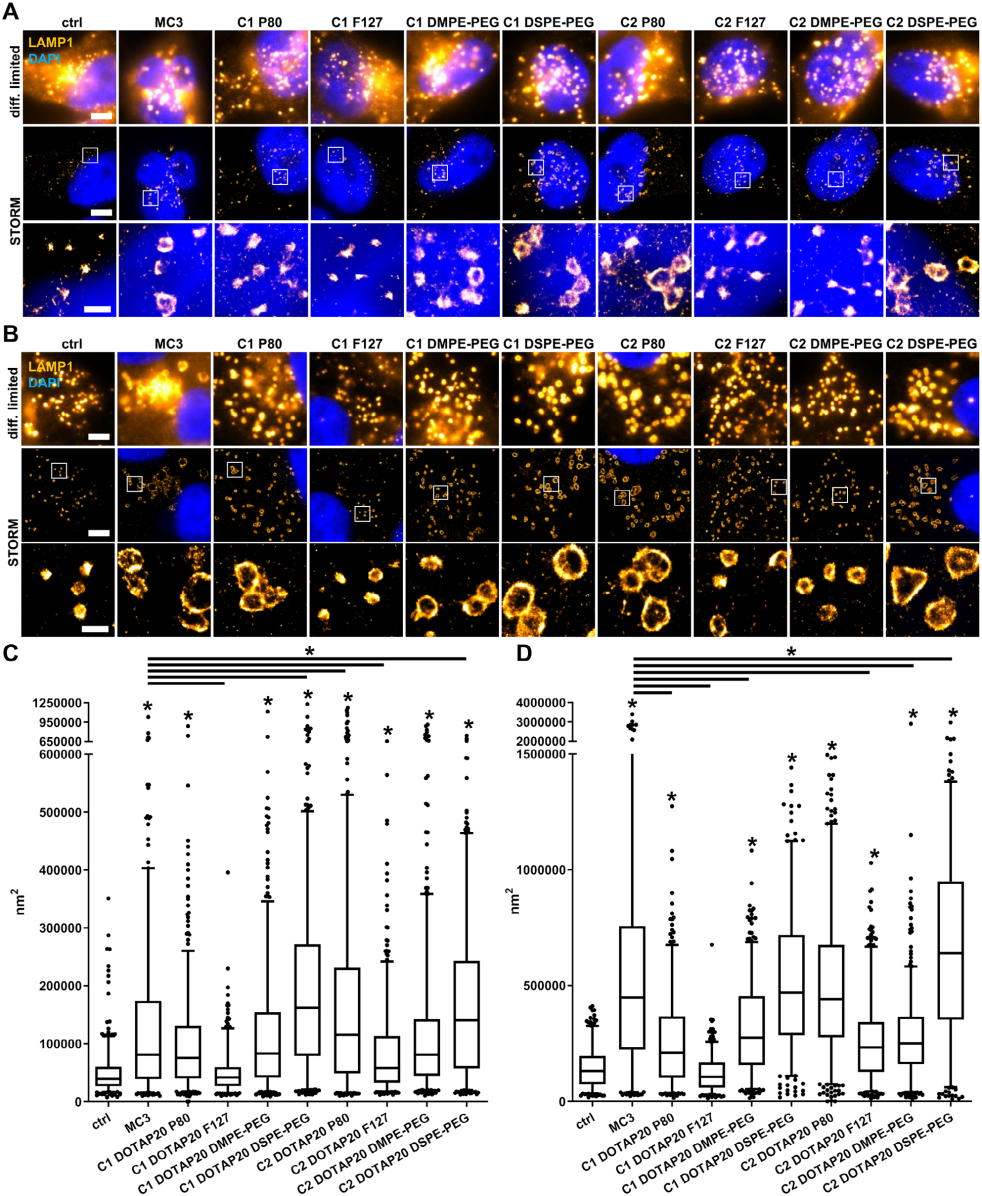
LNPs induce lysosome swelling in both HeLa and Huh7 cells treated with MC3, C1 DOTAP, and C2 DOTAP LNPs. A,B) Representative images of HeLa and Huh7 lysosomes, respectively after 24 h of LNP cell treatment. Anti‐LAMP1 staining (yellow), nuclei stained with DAPI (blue). Scale bars: diffraction‐limited and full view STORM 5 µm, zoom‐in STORM 1 µm. C,D) Quantification of lysosome sizes in HeLa and Huh7 cells, respectively, with a deep learning method. *p < 0.05 compared to the ctrl unless otherwise indicated. Kruskal‐Wallis test with Dunn's post hoc correction. The box extends from the 25^th^ to 75^th^ percentile, the line in the middle of the box represents the median and the whiskers show 5 and 95 percentiles. The number of images and identified objects (lysosomes) in the quantification are indicated in Tables S1 and S2 (Supporting Information).

As shown in **Figure** **9** all the LNPs, except the C1 F127 ones, induced lysosomal swelling compared to the untreated control cells in both cell lines. Although the functional LNPs (MC3 in both cell lines and C2 DOTAP20 P80 in HeLa cells) were among the ones causing the biggest lysosome size changes, no clear correlation could be seen between the LNP performance and the effect on lysosome morphology. Similar trends were already detectable at 4 h time point in HeLa cells, although at this point the lysosomal swelling was less pronounced (Figure S11, Supporting Information). Of note, the lysosomes in Huh7 cells were overall clearly bigger than in HeLa cells. Lysosome size has been previously linked to endosomal escape efficiency, with smaller lysosomes being more prone to releasing the cargo.^[^ ^71^ ^]^ This might be one factor contributing to the poor overall LNP performance in Huh7 cells. Interestingly, the size distribution of lysosomes in all our samples was wide, suggesting that only a fraction of the lysosomes are affected by the LNP treatment. Previously it has been shown that only < 2 % of the oligonucleotide cargo manages to escape endosomes upon LNP‐mediated delivery,^[^ ^72^ ^]^ which is further supported by the results of Vermeulen et al. who demonstrated that in their polyplex pDNA delivery system only < 10 % of HeLa endosomes show endosomal escape.^[^ ^71^ ^]^ Our results suggest that in addition to the extensive endosomal entrapment, the endolysosomal machinery is not fully exploited for the LNP‐mediated cargo delivery. One other potential mechanism is lipid exchange between the LNPs and the lysosomal lipid membrane inside the lysosome, which leads to lysosomal swelling but not rupture.

Lysosomal swelling is often connected to the so‐called “proton sponge” effect, where a compound or material inside the lysosomes can accept protons, which leads to excess proton flow to facilitate the acidification, and consequent osmotic swelling leading to lysosome rupture and cargo release.^[^ ^73^, ^74^ ^]^ In contrast to ILs, DOTAP is positively charged independently of the pH, which does not support the osmotic swelling theory behind the observed lysosome size increase. Based on endosome acidification inhibition studies, IL‐containing LNPs do not induce increased proton flow either.^[^ ^72^ ^]^ Interestingly, since non‐functional LNPs can cause similar or even more pronounced swelling compared to functional ones, this swelling must happen without cargo release, suggesting that the lysosome membrane stays intact and unruptured. LNP fusion with endosomal membranes, which has been shown to occur with highly structured LNPs,^[^ ^5^, ^18^, ^21^, ^36^ ^]^ could also increase the lysosome membrane area and contribute to swelling, but such fusion tends to lead to cargo release, which was not the case with most of our LNPs.

Lysosome swelling could be also caused by LNP accumulation in these compartments leading to “stretching” of the structures. Some studies suggest that cargo release happens mainly from the early endosomal compartments^[^ ^4^, ^65^ ^]^ and considering that the endosomal escape rate tends to be small, it is possible that LNPs accumulate in the later LAMP1‐positive compartments. Still, there is evidence that IL‐containing LNPs fuse equally well with both early and late endosomal model membranes,^[^ ^75^ ^]^ and oligonucleotide cargo can escape from the late endosomes in addition to the earlier ones,^[^ ^72^ ^]^ suggesting that more work is needed to elucidate the complex trafficking process in different cell systems. However, this was not within the scope of the present study. In the case of the SSO cargo used in this work, which is more stable compared to many other RNA species, the escape from the more acidic later endosomal compartments might not compromise its successful delivery.

## 3 Conclusion

This work aimed to shed light on the role of lipid phase structures on LNP performance by designing LNPs using a series of compositionally unique and highly similar lipid mixtures but different phase behaviors, which were then combined with CL / IL and a stabilizer to form LNPs. This approach enabled us to minimize effects due to composition differences between LNPs and focus on the differences in structure between the LNPs. Based on our screen, we identified a novel untraditional LNP composition, which outperformed the Onpattro composition standard LNPs being less cytotoxic and delivering the SSO cargo more efficiently and with a shorter timescale into HeLa cells. Unlike neutral Onpattro composition particles, these LNPs incorporated a CL DOTAP. Unexpectedly, the base structure of the best‐performing LNPs had an inverse micellar phase (Fd3m) organization, and the ones having an inverted hexagonal phase (H_II_) did not demonstrate any endosomal escape. This suggests that hexagonal phase formation is not required or sufficient for functional oligonucleotide delivery and that other highly curved lipid phases can facilitate oligonucleotide delivery. Whilst some studies have indicated this may be the case, our approach to vary the structure merely by varying lipid ratios rather than the chemical composition of the lipids, enables us to draw structure function conclusions in a way that to the best of our knowledge has not been possible in other studies. Our results also highlight the key role of the stabilizer choice on the LNP performance, showing that in our LNP formulation, P80 can outperform the commonly used PEGylated lipids DMPE‐PEG2000 and DSPE‐PEG2000. Finally, we detected that irrespective of LNP performance or cell uptake, almost all LNP induced lysosomal swelling, supporting previous observations about the complexity of endosomal escape and overall mechanistic features of LNP‐mediated drug delivery. In summary, our approach of varying LNP structure by altering the ratios of the constituent lipids, combined with nanoscale structure studies and biological readouts of LNP performance, has enabled us to demonstrate that it is feasible to study structure‐function relationships without altering lipid structural composition and that many highly curved lipid phases could facilitate enhanced delivery and outperform gold standard formulations. Moving forward, there is a rich lipidic and structural landscape to explore using our approach which in the long term could enable structure‐function correlations for LNPs.

## 4 Experimental Section

### Materials

alamarBlue Cell Viability Reagent (Invitrogen, Thermo Fisher Scientific, Cat. No. DAL1100), Alexa Fluor 647 Phalloidin (Invitrogen, Thermo Fisher Scientific, Cat. No. A22287), Bovine Serum Albumin Protease‐free Powder (Fisher Scientific, Product No. BP9703‐100), Catalase from bovine liver (Merck, Product No. C1345), T‐75 Cell culture flask (Sarstedt, Product No. 83.3911.002), Chloroform 99.0%–99.4% stabilized (VWR, Cat. No. 22 711.324), Citric acid (Merck, Product No. C1857), Cysteamine (Merck, Product No. 30 070), Cytiva Mini Dialysis Kit (Cytiva, Product No. 80‐6483‐75, DAPI (4′,6‐Diamidino‐2‐Phenylindole, Dihydrochloride) (Invitrogen, Thermo Fisher Scientific, Cat. No. D1306), D(+)‐Glucose anhydrous (Scharlau, Ref. GL01251000), Donkey anti‐Rabbit IgG (H+L) Highly Cross‐Adsorbed Secondary Antibody Alexa Fluor 647 (Invitrogen, Thermo Fisher Scientific, Cat. No. A31573, lot 1 693 297), Dulbecco's modified Eagle's medium (DMEM) with high glucose & GlutaMAX (Gibco, Thermo Fisher Scientific, Cat. No. 31966‐021), Dulbecco's Phosphate‐Buffered Saline (DPBS) no calcium no magnesium (Gibco, Thermo Fisher Scientific, Cat. No. 14 190 094), Falcon 96‐Well Cell Culture‐Treated Flat‐Bottom Microplate (Fisher Scientific, Product Code 10 088 612), Fetal Bovine Serum (FBS; Gibco, Thermo Fisher Scientific, Cat. No. 10270‐106), Glass vials (2 mL, low adsorption; Merck, Product No. 29651‐U), Glucose Oxidase from *Aspergillus niger* (Merck, Product No. G7141), 25 % Glutaraldehyde Aqueous Solution (Merck, Product No. 1.04239.0250), LAMP1 (D2D11) XP Rabbit mAb (Cell Signaling Technology, Cat. No. 9091S, lot: 6), Lipofectamine 2000 Transfection Reagent (Invitrogen, Thermo Fisher Scientific, Cat. No. 11 668 027), Luciferase Assay System (Promega, Cat No. E1501), µ‐Plate 96 Well Black (Ibidi, Cat. No. 89 626), µ‐Slide 8 Well Glass Bottom (Ibidi, Cat. No. 80 827), 20 % Paraformaldehyde Aqueous Solution (Electron Microscopy Sciences, Cat. No. 15 713), Penicillin‐Streptomycin 10 000 U mL (P/S, Gibco, Thermo Fisher Scientific, Cat. No. 15 140 122), Pierce BCA Protein Assay Kit (Thermo Fisher Scientific, Cat No. 23 227), Quant‐it RiboGreen RNA Assay Kit (Invitrogen, Thermo Fisher Scientific, Cat. No. R11490), Sodium Chloride (Merck, Product No. S7653), Sodium phosphate dibasic (Merck, Product No. S7907), Triton X‐100 (Merck, Product No. X100), Trizma base (Merck, Product No. T6066), TrypLE Express Enzyme (1X) (Gibco, Thermo Fisher Scientific, Cat. No. 12 604 039), 96 well Cell culture plate (Sarstedt, Product No. 83.3924), 96 Well White Plate (Thermo Fisher Scientific, Cat. No. 236 108).

Luc705‐targeting 18 nucleotides long splice‐switching oligonucleotide (705‐SSO; sequence: 5′‐mC*mC*mU*mC*mU*mU*mA*mC*mC*mU*mC*mA*mG*mU*mU*mA*mC*mA‐3′) was purchased from Integrated DNA Technologies and synthesized with 2′‐O‐methylated (2′‐OMe (m))‐modified bases and phosphorothioate (PS (*))‐saturated backbone linkages. HPLC purification and Na^+^ salt exchange steps were included.

Red blood cells (RBCs) for the hemolysis assay (see Supporting Information for detailed method description and results) were isolated from fresh human blood obtained from Blodcentralen Odenplan, Karolinska University Hospital (ethical permit 2023‐06187‐01, Swedish Ethical Review Authority (Etikprövningsmyndigheten), Modifiering av immunceller).

### Lipids and Stabilizers

1,2‐dimyristoyl‐sn‐glycero‐3‐phosphoethanolamine‐N‐[methoxy(polyethylene glycol)‐2000] (ammonium salt) (DMPE‐PEG2000; Avanti Polar Lipids, Product No. 880 150), 1,2‐dioleoyl‐3‐dimethylammonium‐propane (DODAP; Avanti Polar Lipids, Product No. 890 850), 1,2‐dioleoyl‐sn‐glycero‐3‐phosphocholine (DOPC; provided by Camurus AB), 1,2‐dioleoyl‐3‐trimethylammonium‐propane (chloride salt) (DOTAP; Avanti Polar Lipids, Product No. 890 890), 1,2‐distearoyl‐sn‐glycero‐3‐phosphoethanolamine‐N‐ [methoxy (polyethylene glycol)‐2000] (ammonium salt) (DSPE‐PEG2000; Avanti Polar Lipids, Product No. 880 120), glycerol dioleate (GDO; provided by Camurus AB), Lissamine Rhodamine B 1,2‐Dihexadecanoyl‐*sn*‐Glycero‐ 3‐Phosphoethanolamine, Triethylammonium Salt (rhodamine DHPE) (Invitrogen, Thermo Fisher Scientific, Cat. No. L1392), Pluronic F‐127 (Merck, Product No. P2443), Polysorbate 80 (P80; provided by Camurus AB).

### Methods—Lipid Nanoparticle Formulation

Lipids were weighed and dissolved in chloroform at 5–10 mg mL^−1^ concentrations. Lipid solutions were mixed in 2 mL glass vials in mol‐% ratios indicated in Table 1, with a total lipid amount of 1.35 µmol (0.9–1.0 mg) per vial. Due to the higher concentration requirement, films of four times this amount were prepared for CryoEM. Chloroform was evaporated overnight in a fume hood at room temperature to give homogeneous lipid films, which were stored and sealed in a nitrogen atmosphere at −20 °C until LNP formulation. DMPE‐PEG2000 and DSPE‐PEG2000 stabilizers (stock concentrations 1 mg mL^−1^) were added afterward (see mol‐% in Table 1). For the uptake imaging experiments, some of the films were supplemented with 0.1 mol‐% Rhodamine B lipid (stock concentration 0.1 mg mL^−1^ in chloroform).

To formulate the LNPs lipid films were hydrated in 5 µL absolute ethanol and 65 µL 8 mM citric acid + 4 mM dibasic sodium phosphate + 150 mM NaCl at pH 3 (referred to as pH 3 citrate phosphate buffer from now on). Samples were subsequently heated on a 60 °C hot plate for 30 min, followed by the addition of freshly prepared P80 and F‐127 stabilizer solutions in pH 3 citrate phosphate buffer (stock concentrations 3.36 and 0.79 mg mL^−1^, respectively) to give the mol‐% indicated in Table 1. In the case of DMPE‐ and DSPE‐PEG2000 samples a corresponding volume of buffer was added. 370 µL SSO stock (13.51 nmol mL^−1^, in pH 3 citrate phosphate buffer) was added to all the samples to reach a final concentration of 10 000 nM (N/P ratio 3). The samples were sonicated with a Vibra‐Cell tip sonicator (Sonics & Materials) using pulse mode (1 s on, 1 s off, 30 % amplitude for 2–3 × 5 min), followed by 30 s of vortexing. Residual titanium from the sonication tip was removed via 4000 rpm centrifugation for 10 min in an Eppendorf benchtop centrifuge, after which the samples were transferred to new glass vials and stored at room temperature until used for the various experiments within 2–3 days.

DLin‐MC3‐DMA control LNPs (called here MC3 LNPs) were formulated in AstraZeneca using a NanoAssemblr microfluidic system (Precision NanoSystems). In brief, lipids were prepared in ethanol at a mol‐% ratio of 50:38.5:10:1.5 (MC3:cholesterol:DSPC:DMPE‐PEG2000). SSO cargo was prepared in a 50 mM citrate pH 3 buffer (TekNova, Product No. Q2445). Lipid and SSO‐containing solutions were mixed at an ethanol: aqueous ratio of 3 at a constant flow rate of 12 mL min^−1^, the N/P ratio being 3. Particles were dialysed overnight with a 3 kDa MWCO dialysis cassette (Thermo Fisher Scientific, Cat. No. 87 729) into 20 mM Tris buffer pH 7.4 at 4 °C, followed by 0.2 µm filtration (Acrodisc, Pall Corporation, Product No. 4602) and concentration with a 30 000 MWCO Amicon Ultra centrifugal spin column (Merck Millipore, Cat. No. UFC803096) at 3000 g for 20 min at 4 °C. The volume was recorded after every processing step and the SSO concentration in the final product was determined by back‐calculation knowing the initial concentration. MC3 LNPs were aliquoted and stored at –80 °C in 20 mM Tris buffer pH 7.4 containing 8 % sucrose.

### Small Angle X‐ray Scattering

Small‐angle X‐ray scattering was performed either using a benchtop or synchrotron source. The benchtop source was an Anton Paar SAXSpoint 2.0 instrument located at the Research Institutes of Sweden (RISE) which consists of a Microsource Supernova Copper source of wavelength 1.541 Å coupled with a 2D Eiger R 1M detector. Samples were run at 25, 37 °C under vacuum using a sample holder with Kapton windows. The SAXS set up used a sample‐to‐detector distance of 0.1091 m which enabled measurements over a q range of 0.0388 < *q* < 39.9800 Å^−1^ where q = (4π/λ)sin(θ/2) and data was acquired for 150 s per sample. The tiff files generated from samples measured on the benchtop source were radially integrated using Axcess an in‐house software.^[^ ^76^ ^]^ Samples measured using a synchrotron were measured at Diamond Light Source on beamline I22^[^ ^77^ ^]^ or at MAXIV on the CoSAXS beamline. At Diamond, all samples were measured in SAXS / WAXS mode using Pilatus 2M detectors and camera lengths of 4.28 m, which correspond to a q range of 0.00513 < *q* < 0.590 Å^−1^ using an energy of 18 keV. Samples were measured at the synchrotron using polycarbonate capillaries (Spectrum Plastics, US) or 1.5 mm borosilicate capillaries (Capillary Tube Supplies Ltd, UK) at temperatures specified in each dataset (25, 37 °C) and exposure times of 50 ms. At MAXIV all samples were measured in SAXS / WAXS mode using the SAXS Eiger2 4M and WAXS Pilatus L‐shaped 2M Mythen 12 detectors with a camera length of 2.5, which corresponds to a q range of 0.00503 < *q* < 0.280 Å^−1^ using an energy of 12.4 keV.

In all cases, samples were prepared using the relevant molar ratios of lipids using 10–25 mg total of lipids for each sample. Lipids were co‐dissolved in chloroform, dried to form a lipid film, and stored under nitrogen at −20 °C as described in the LNP preparation method. For SAXS measurement, these films were hydrated in either MQ water, pH 3 citrate phosphate buffer, or pH 7.4 citrate phosphate buffer (described previously) and hydrated at 80–90 wt‐%. Samples were freeze‐thaw cycled between −80 and 65 °C a minimum of ten times to ensure a homogeneous distribution of solvent and transferred to the relevant sample holder for measurement.

SAXS data was analyzed using AXcess.^[^ ^76^ ^]^ Briefly, the 2D SAXS images were radially integrated to give 1D diffraction patterns. The Bragg peaks were then fitted and indexed using characteristic peak spacings from known lipid structures to extract the d spacings. For the inverted hexagonal H_II_ phase d spacings were converted to lattice parameters as described previously.^[^ ^78^ ^]^

### Small Angle Neutron Scattering

SANS experiments were performed on the ZOOM beamline at ISIS Neutron and Muon Source, Rutherford Appleton Laboratory, Didcot, UK using a sample changer with Julabo water bath for temperature control, 1 mm pathlength quartz cuvette cells and sample volumes of 150 µL. LNP samples were prepared in pH 3 citrate phosphate buffer (described previously) and diluted to 1.35 mg mL^−1^ by diluting 40 µL into deuterated buffer to give a final pH of either pH 5 or pH 7.4 deuterated citrate phosphate buffers and a final D:H ratio of 91% deuterium in the buffer, to reduce incoherent scattering from H_2_O and provide good contrast between the lipids and the buffer. Samples were loaded into 1 mm Hellma cells for SANS measurements at 25 and 37 °C. Samples were measured for 8 µA (SANS) and 5 µA (TRANS) proton current using a beamline configuration of L1 = L2 = 4 m collimation and sample‐detector distances to give a scattering vector q = (4π/λ)sin(θ/2) range of 0.00416–0.77484 Å^−1^, where θ is the scattering angle and neutrons of wavelengths λ of 1.75–16.5 Å were used simultaneously by time of flight. Data reduction and background subtraction was performed using MantidPlot and scattering data was fitted using SASView v 4.2.2.^[^ ^79^ ^]^ Samples were fitted using either a Power Law or Unified Model fit over defined q ranges which are defined for the relevant fits. Tables including all fitted parameters and errors in the fits (including 𝜒^2^ values) are included in the supplementary information Tables S3 and S4 (Supporting Information).

### Dynamic Light Scattering

All the LNPs were characterized by dynamic light scattering (DLS; Zetasizer Nano ZS, Malvern) in both PBS and pH 3 citrate phosphate buffer, diluted 1:50 (v/v).

### Cryo‐Electron Microscopy

The LNP structure was visualized in both pH 3 citrate phosphate buffer and at pH 7 (PBS) with CryoEM. To increase the pH of the samples to 7 a dialysis with 1 kDa cut‐off (Cytiva Mini Dialysis Kit) was conducted for 24–48 h at room temperature in PBS. The pH was checked after dialysis with pH paper. For the CryoEM 3.0 µL of the sample was applied to glow‐discharged Quantifoil R2/2 grids on 400 copper mesh (Quantifoil Micro Tools GmbH) with an additional thin continuous carbon layer on top. Frozen‐hydrated specimens were prepared with an automatic plunge freezer FEI Vitrobot (Thermo Fisher Scientific) operated at 16 °C and 100 % relative humidity. The samples were incubated 10 s on the grids, blotted for 3 s, and plunged into liquid ethane. The vitrified specimens were imaged at JEOL JEM‐2100f transmission electron microscope (JEOL Ltd.) using TVIPS TemCam‐XF416 CMOS camera (Tietz Video and Image Processing Systems GmbH) and nominal magnifications of 25000x and 30000x.

### Cargo‐Loading Assay

To measure the loading efficiency of SSO cargo, an RNA‐detecting RiboGreen assay was conducted for the LNPs before and after lysing them with 2 vol‐% Triton X‐100. Briefly, samples were diluted in the assay buffer, pipetted in 96‐well imaging microplates (Falcon, Corning) in duplicates, followed by Triton X‐100 treatment of one of the duplicate wells for 10 min at 37 °C (the other well was treated with assay buffer without Triton X‐100). Standard samples at concentrations of 3.05, 1.52, 0.31, 0.04, and 0.00 µg mL^−1^, prepared from the SSO stock, were included in all the plates and treated similarly to the LNP samples with/without Triton X‐100. RiboGreen reagent, diluted in the assay buffer 1:200, was added to the wells in 1:1 vol. ratio and the fluorescence was immediately measured at 480/520 nm with a plate reader.

### Cell Culture

HeLa Luc705 and Huh7 Luc705 cells were used to screen and characterize the SSO‐loaded LNPs.^[^ ^27^, ^80^ ^]^ They have a stable expression of a plasmid carrying a Luc coding sequence interrupted by an insertion of intron 2 from β‐globin pre‐mRNA. This intron has a cryptic splice site. Unless the aberrant splice site is masked by SSO, the pre‐mRNA of Luc will be incorrectly processed. Thus, by using these cells, the LNP‐mediated functional delivery of SSO can be evaluated by measuring Luc activity.

Cells were grown at 37 °C, 5 % CO_2_ in 95 % humidity in T75 cell culture flasks. The cells were detached with TrypLE reagent and passaged twice a week.

### Cell Treatment with the LNPs

For the various experiments, a cell density of 300–500 cells mm^−2^ was used. Cells were seeded a day before the LNP treatment. LNPs were dosed based on the SSO concentration in the samples and the following doses were used: 100, 500, and 1000 nM SSO. Before feeding them to cells, LNPs were incubated in the serum‐containing cell culture medium for 3 Lh in the cell culture incubator to allow for the protein corona formation. A pH 3 citrate phosphate buffer control was included where the volume of this buffer corresponding to the highest volume of LNP sample used (1000 nM dose) was mixed with the cell culture medium. Moreover, Lipofectamine 2000 transfection control was conducted following the manufacturer's protocol. Here, only a 100 nM SSO dose was used since with higher doses Lipofectamine 2000 is highly cytotoxic.

### Cytotoxicity Assay

The cytotoxicity of the novel LNPs as well as the control groups was evaluated at 4 and 24 h with alamarBlue assay. Briefly, the cells were cultured on Falcon 96‐Well Cell Culture‐Treated Flat‐Bottom Microplates with triplicate wells of each condition. At the time point, 1:10 (v/v) Alamar blue reagent was added to the wells and incubated for 1 h in the cell culture incubator. The fluorescence was measured at 560/590 nm with Varioskan LUX Multimode Microplate Reader (Thermo Fisher Scientific). Three independent experiments were conducted with both cell lines.

### Luciferase Assay and Total Protein Quantification

To evaluate the LNP‐mediated functional delivery of the SSO cargo, Luc enzyme activity was determined after treating the samples with the LNPs for 4 and 24 h. At each time point, the medium was removed and the cells lysed with 0.1 vol‐% Triton X‐100 in PBS. To facilitate the lysis, one freeze‐thaw cycle (−80 °C) was conducted. Luciferase enzyme activity assay was conducted in white 96‐well plates. 30 µL lysate was mixed with an equal volume of Luc assay reagent prepared according to the manufacturer's protocol. Luminescence was measured immediately with a Varioskan LUX Multimode Microplate Reader (Thermo Fisher Scientific). Three independent experiments were conducted with both cell lines.

Luciferase activity was normalized with the total protein in the samples, determined with BCA assay from the same lysates used for the Luc activity measurement. Albumin standards, prepared according to the kit protocol, were included in all the plates. 15 µL of samples and standards were pipetted into 96 wells and 200 µL of working solution (50:1 ratio of kit solutions A and B) was added to the wells. The plates were incubated at +37 °C for 1 h before measuring the absorbance at 562 nm with Varioskan LUX Multimode Microplate Reader (Thermo Fisher Scientific).

### Uptake Imaging

To evaluate the uptake of the novel LNPs into cells, Rhodamine B labeled (0.1 mol‐%) LNPs were used for the cell treatment. Cells were cultured on Ibidi 96‐well black µ‐plates, with duplicate wells of each condition, treated with the LNPs, and fixed at 1, 4, and 24 h. At each time point, the samples were thoroughly washed with PBS and fixed with a solution of 4 vol‐% paraformaldehyde (PFA) and 0.2 vol‐% glutaraldehyde (GA) for 15 min at room temperature (RT). The samples were then blocked with 3 vol‐% bovine serum albumin (BSA) for 30 min at RT and counterstained with Alexa Fluor 647 phalloidin (diluted 1:500 in 3 vol‐% BSA; 30 min at RT) and DAPI (diluted 1:5000 in PBS; 5 min at RT). The samples were stored at 4 °C until the imaging. Imaging was conducted with ImageXpress Micro high‐throughput microscope (MolecularDevices) using a 20x objective, DAPI, CY5, and CY3 fluorescent channels, and imaging four regions of interest in each well.

### Immunocytochemical Staining

To evaluate the structural changes in late endosomes / lysosomes in response to LNP treatment, a LAMP1 immunocytochemical staining, followed by STORM super‐resolution microscopy, was conducted as described in.^[^ ^73^ ^]^ Briefly, cells were cultured on Ibidi 8‐well glass bottom µ‐slides and treated with LNPs (500 nM SSO dose) for 24 h. Cells were fixed with a solution of 4 vol‐% PFA and 0.2 vol‐% GA for 15 min at RT, permeabilized with 0.05 vol‐% Triton X‐100 for 5 min at RT, followed by 1.5 h blocking with 3 vol‐% BSA and overnight incubation of anti‐LAMP1 primary antibody at 4 °C (diluted 1:200 in 3 vol‐% BSA). Alexa Fluor 647 conjugated secondary antibody was diluted 1:1000 in 3 vol‐% BSA and incubated at RT for 1.5 h. To fix the antibody proteins a 10 min post‐fixation with 2 vol‐% PFA was performed. Nuclei were stained with DAPI (diluted 1:5000 in PBS, 5 min incubation at RT). The samples were stored at 4 °C until the STORM imaging.

### Stochastic Optical Reconstruction Microscopy–Imaging

Immediately before imaging, the sample was soaked in imaging buffer with the following composition: Tris buffer (160 mM Tris, 40 mM NaCl, pH adjusted to 8.0), 10 wt‐% glucose, 0.5 mg mL^−1^ glucose oxidase, 47 µg mL^−1^ catalase and 10 mM cysteamine. The slide was sealed with parafilm to prevent oxygen entry. Stochastic optical reconstruction microscopy was conducted with Nikon Ti Eclipse inverted microscope (Nikon), housing cube filters (excitation: Chroma ZET405/488/561/640x, emission: Chroma ZET405/488/561/640m) and TIRF dichroic ZET405/488/561/640 bs and equipped with Cairn laser module (Cairn Research) with 300 mW 405 nm and 140 mW 642 lasers used here. CFI SR Apo TIRF 100x oil objective (N.A. 1.49) was used in combination with 1.5x Optovar lens, giving a final magnification of 150x. The camera (Andor iXON Ultra 888 EMCCD, Oxford Instruments) had a pixel size of 13 µm, resulting in a final pixel size of 87 nm with 150x magnification. The image acquisition was controlled with MetaMorph (Molecular Devices) and Micro‐Manager open‐source software.^[^ ^81^ ^]^ The region of interest (ROI) was set to 256×256 pixels. To serve as a reference, a widefield diffraction‐limited image was taken from each ROI before starting the STORM acquisition. For the STORM acquisition, the laser power was increased to 100 %. The acquisition was only started when an optimal level of fluorophore photoswitching was reached (visually evaluated by the user). Around 30 000 frames, with an exposure time of 30 ms/frame and electron‐multiplying gain of 100, were recorded for each image. DAPI images were acquired only in the diffraction‐limited mode.

The images were reconstructed with the ThunderSTORM plugin in Fiji^[^ ^82^ ^]^ and drift‐corrected using the ThunderSTORM cross‐correlation algorithm. Images were filtered based on the sigma values (standard deviation of the Gaussian fit over the point spread function of each blink) to remove low‐quality signals (e.g., noise and partially overlapping fluorophores). Moreover, additional intensity‐based filtering was conducted to further remove background and noise and thus enable higher‐quality feature detection in the lysosome quantification. The reconstruction and post‐processing parameters are described in more detail in Supplementary information. All the images were treated identically and visualized with the Normalized Gaussian visualization method using a magnification of 10 and an image‐specific uncertainty constant. This gives a final pixel size of 8.7 nm in the STORM visualizations.

### Stochastic Optical Reconstruction Microscopy–Quantification

Lysosome sizes from the STORM visualizations were quantified with a deep learning technique. HeLa and Huh7 STORM data were treated separately due to the differences in lysosome appearance between these cell types, which might have decreased the quality of a general model. A training dataset for a deep learning model to identify lysosomes from the STORM images consisted of a 10‐image subset of images with manually annotated lysosomes (annotations done in Fiji).^[^ ^83^ ^]^ These images were converted to mask images using the ROI map function of the LOCI plugin. The training was done with the dataset of 10 paired original STORM images and manually annotated images (image dimensions: original STORM images 2560×2560 pixels, patch size: see **Table** **2**) with a mae loss function, using the StarDist 2D ZeroCostDL4Mic notebook (v 1).^[^ ^68^, ^69^, ^70^ ^]^ The parameters for the training are indicated in Table 2. The model was retrained from a pretrained model. Key python packages used included tensorflow (v0.1.12), Keras (v2.3.1), csbdeep (v0.6.3), numpy (v1.19.5), cuda (v11.1.105 Build cuda_11.1.TC455_06.29190527_0). The training was accelerated using a Tesla T4/K80 GPU. The quality of the trained model was evaluated with 10 validation images not used for the training (manually annotated the same way as the training data). The areas of objects identified with the deep learning model were determined with the software package CellProfiler (version 3.1.9., Broad Institute).^[^ ^84^, ^85^ ^]^ For the 4 h STORM data the model created with the 24 h HeLa data was used.

**Table 2:** Training parameters for the deep learning model were used to quantify the lysosome areas from the STORM images.

Cell Type	n of Epochs	Patch Size	Batch Size	Number of Steps	Validation Percentage [During Training]	n Rays	Grid Parameter	Initial Learning Rate	Augmentation Factor
HeLa	150	256 × 256 pixels	4	1800	20	32	2	0.0003	6
Huh7	100	272 × 272 pixels	4	1584	20	32	2	0.0003	6

### Statistical Analyses

Statistical analyses were performed with Graphpad Prism 5.0. All the quantitative data are shown as means and standard deviations. Statistical significance was determined using the nonparametric Kruskall‐Wallis test combined with Dunn's post hoc multiple comparison test. The statistical significance level was set to *p* < 0.05. SANS / SAXS errors are described separately as the statistical analysis was not feasible. For these datasets, the errors represent the error in the fit for individual datasets.

## Conflict of Interest

M.M.S. has invested in, consulted for (or was on scientific advisory boards or boards of directors), and conducts sponsored research funded by companies related to the biomaterials field. M.M.S. is an advisor to and has equity in Nanovation Therapeutics. All other authors declare they have no competing interests. The lipids 1,2‐dioleoyl‐sn‐glycero‐3‐phosphocholine (DOPC) and glycerol dioleate (GDO) were provided by Camurus AB.

## Supporting information



Supporting Information

## Data Availability

Raw research data is available upon request from the corresponding author.

## References

[1] X. Hou , T. Zaks , R. Langer , Y. Dong , Nat. Rev. Mater. 2021, 6, 1078. 34394960 10.1038/s41578-021-00358-0 PMC8353930

[2] Y. Suzuki , H. Ishihara , Drug Metab. Pharmacokinet. 2021, 41, 100424. 34757287 10.1016/j.dmpk.2021.100424 PMC8502116

[3] A. Akinc , M. A. Maier , M. Manoharan , K. Fitzgerald , M. Jayaraman , S. Barros , S. Ansell , X. Du , M. J. Hope , T. D. Madden , B. L. Mui , S. C. Semple , Y. K. Tam , M. Ciufolini , D. Witzigmann , J. A. Kulkarni , R. van der Meel , P. R. Cullis , Nat. Nanotechnol. 2019, 14, 1084. 31802031 10.1038/s41565-019-0591-y

[4] P. Paramasivam , C. Franke , M. Stöter , A. Höijer , S. Bartesaghi , A. Sabirsh , L. Lindfors , M. Y. Arteta , A. Dahlén , A. Bak , S. Andersson , Y. Kalaidzidis , M. Bickle , M. Zerial , J. Cell Biol. 2022, 221, 202110137. 10.1083/jcb.202110137 PMC8666849 34882187

[5] J. Philipp , A. Dabkowska , A. Reiser , K. Frank , R. Krzysztoń , C. Brummer , B. Nickel , C. E. Blanchet , A. Sudarsan , M. Ibrahim , S. Johansson , P. Skantze , U. Skantze , S. Östman , M. Johansson , N. Henderson , K. Elvevold , B. Smedsrød , N. Schwierz , L. Lindfors , J. O. Rädler , Proc. Natl. Acad. Sci. U.S.A. 2023, 120, 2310491120. 10.1073/pnas.2310491120 PMC10723131 38055742

[6] M. Yanez Arteta , T. Kjellman , S. Bartesaghi , S. Wallin , X. Wu , A. J. Kvist , A. Dabkowska , N. Székely , A. Radulescu , J. Bergenholtz , L. Lindfors , Proc. Natl. Acad. Sci. U.S.A. 2018, 115, E3351. 29588418 10.1073/pnas.1720542115 PMC5899464

[7] M. Verma , I. Ozer , W. Xie , R. Gallagher , A. Teixeira , M. Choy , Nat. Rev. Drug Discov. 2023, 22, 349. 36627441 10.1038/d41573-023-00002-2

[8] A. Del Grosso , M. Galliani , L. Angella , M. Santi , I. Tonazzini , G. Parlanti , G. Signore , M. Cecchini , Sci. Adv. 2019, 5. 10.1126/sciadv.aax7462 PMC6867879 31799395

[9] R. Kedmi , N. Veiga , S. Ramishetti , M. Goldsmith , D. Rosenblum , N. Dammes , I. Hazan‐Halevy , L. Nahary , S. Leviatan‐Ben‐Arye , M. Harlev , M. Behlke , I. Benhar , J. Lieberman , D. Peer , Nat. Nanotechnol. 2018, 13, 214. 29379205 10.1038/s41565-017-0043-5

[10] S. Liu , Q. Cheng , T. Wei , X. Yu , L. T. Johnson , L. Farbiak , D. J. Siegwart , Nat. Mater. 2021, 20, 701. 33542471 10.1038/s41563-020-00886-0 PMC8188687

[11] Q. Cheng , T. Wei , L. Farbiak , L. T. Johnson , S. A. Dilliard , D. J. Siegwart , Nat. Nanotechnol. 2020, 15, 313. 32251383 10.1038/s41565-020-0669-6 PMC7735425

[12] J. G. Rurik , I. Tombácz , A. Yadegari , P. O. Méndez Fernández , S. V. Shewale , L. Li , T. Kimura , O. Y. Soliman , T. E. Papp , Y. K. Tam , B. L. Mui , S. M. Albelda , E. Puré , C. H. June , H. Aghajanian , D. Weissman , H. Parhiz , J. A. Epstein , Science 2022, 375, 91. 34990237 10.1126/science.abm0594 PMC9983611

[13] K. Paunovska , C. J. Gil , M. P. Lokugamage , C. D. Sago , M. Sato , G. N. Lando , M. Gamboa Castro , A. V. Bryksin , J. E. Dahlman , ACS Nano 2018, 12, 8341. 30016076 10.1021/acsnano.8b03640 PMC6115295

[14] S. Sabnis , E. S. Kumarasinghe , T. Salerno , C. Mihai , T. Ketova , J. J. Senn , A. Lynn , A. Bulychev , I. McFadyen , J. Chan , Ö. Almarsson , M. G. Stanton , K. E. Benenato , Mol. Ther. 2018, 26, 1509. 29653760 10.1016/j.ymthe.2018.03.010 PMC5986714

[15] R. Pattipeiluhu , G. Arias‐Alpizar , G. Basha , K. Y. T. Chan , J. Bussmann , T. H. Sharp , M. Moradi , N. Sommerdijk , E. N. Harris , P. R. Cullis , A. Kros , D. Witzigmann , F. Campbell , Adv. Mater. 2022, 34, 2201095. 10.1002/adma.202201095 PMC9461706 35218106

[16] Y. Wu , Y. Xiong , L. Wang , Q. Zhou , L. Li , P. A. Levkin , G. Davidson , L. Gao , W. Deng , Biomater. Sci. 2020, 8, 3021. 32322846 10.1039/d0bm00331j

[17] Y. Wu , L. Wang , Y. Xiong , Q. Zhou , L. Li , G. Chen , Y. Ping , G. Davidson , P. A. Levkin , L. Gao , W. Deng , Acta Biomater. 2020, 115, 410. 32853811 10.1016/j.actbio.2020.08.029

[18] M. Hammel , Y. Fan , A. Sarode , A. E. Byrnes , N. Zang , P. Kou , K. Nagapudi , D. Leung , C. C. Hoogenraad , T. Chen , C.‐W. Yen , G. L. Hura , ACS Nano 2023, 17, 11454. 37279108 10.1021/acsnano.3c01186 PMC10311593

[19] S. C. Semple , A. Akinc , J. Chen , A. P. Sandhu , B. L. Mui , C. K. Cho , D. W. Y. Sah , D. Stebbing , E. J. Crosley , E. Yaworski , I. M. Hafez , J. R. Dorkin , J. Qin , K. Lam , K. G. Rajeev , K. F. Wong , L. B. Jeffs , L. Nechev , M. L. Eisenhardt , M. Jayaraman , M. Kazem , M. A. Maier , M. Srinivasulu , M. J. Weinstein , Q. Chen , R. Alvarez , S. A. Barros , S. De , S. K. Klimuk , T. Borland , et al., Nat. Biotechnol. 2010, 28, 172. 20081866 10.1038/nbt.1602

[20] L. Zheng , S. R. Bandara , Z. Tan , C. Leal , Proc. Natl. Acad. Sci. U.S.A. 2023, 120, 2301067120. 10.1073/pnas.2301067120 PMC10318962 37364130

[21] B. P. Dyett , H. Yu , J. Strachan , C. J. Drummond , C. E. Conn , Nat. Commun. 2019, 10, 4492. 31582802 10.1038/s41467-019-12508-8 PMC6776645

[22] R. Pattipeiluhu , Y. Zeng , M. M. R. M. Hendrix , I. K. Voets , A. Kros , T. H. Sharp , Nat. Commun. 2024, 15, 1303. 38347001 10.1038/s41467-024-45666-5 PMC10861598

[23] S. L. Yap , H. Yu , S. Li , C. J. Drummond , C. E. Conn , N. Tran , J. Colloid Interface Sci. 2024, 656, 409. 38000253 10.1016/j.jcis.2023.11.059

[24] M. H. Y. Cheng , J. Leung , Y. Zhang , C. Strong , G. Basha , A. Momeni , Y. Chen , E. Jan , A. Abdolahzadeh , X. Wang , J. A. Kulkarni , D. Witzigmann , P. R. Cullis , Adv. Mater. 2023, 35, 2303370. 10.1002/adma.202303370 37172950

[25] Y. Eygeris , S. Patel , A. Jozic , G. Sahay , Nano Lett. 2020, 20, 4543. 32375002 10.1021/acs.nanolett.0c01386

[26] F. Sebastiani , M. Yanez Arteta , M. Lerche , L. Porcar , C. Lang , R. A. Bragg , C. S. Elmore , V. R. Krishnamurthy , R. A. Russell , T. Darwish , H. Pichler , S. Waldie , M. Moulin , M. Haertlein , V. T. Forsyth , L. Lindfors , M. Cárdenas , ACS Nano 2021, 15, 6709. 33754708 10.1021/acsnano.0c10064 PMC8155318

[27] C. S. J. Rocha , K. E. Lundin , M. A. Behlke , R. Zain , S. EL Andaloussi , C. I. E. Smith , Nucleic Acid Ther. 2016, 26, 381. 27629437 10.1089/nat.2016.0631

[28] S. C. Semple , S. K. Klimuk , T. O. Harasym , N. Dos Santos , S. M. Ansell , K. F. Wong , N. Maurer , H. Stark , P. R. Cullis , M. J. Hope , P. Scherrer , Biochimica et Biophysica Acta (BBA) – Biomembranes 2001, 1510, 152. 11342155 10.1016/s0005-2736(00)00343-6

[29] E. Fröhlich , Int J Nanomedicine 2012, 5577. 23144561 10.2147/IJN.S36111 PMC3493258

[30] H. Lv , S. Zhang , B. Wang , S. Cui , J. Yan , J. Controlled Release 2006, 114, 100. 10.1016/j.jconrel.2006.04.014 16831482

[31] M. Jayaraman , S. M. Ansell , B. L. Mui , Y. K. Tam , J. Chen , X. Du , D. Butler , L. Eltepu , S. Matsuda , J. K. Narayanannair , K. G. Rajeev , I. M. Hafez , A. Akinc , M. A. Maier , M. A. Tracy , P. R. Cullis , T. D. Madden , M. Manoharan , M. J. Hope , Angew. Chem., Int. Ed. 2012, 51, 8529. 10.1002/anie.201203263 PMC3470698 22782619

[32] A. K. K. Leung , I. M. Hafez , S. Baoukina , N. M. Belliveau , I. V. Zhigaltsev , E. Afshinmanesh , D. P. Tieleman , C. L. Hansen , M. J. Hope , P. R. Cullis , J. Phys. Chem. C 2012, 116, 18440. 10.1021/jp303267y PMC3434764 22962627

[33] M. Mihailescu , D. L. Worcester , C. L. Carroll , A. R. Chamberlin , S. H. White , Biophys. J. 2023, 122, 1086. 36703558 10.1016/j.bpj.2023.01.031 PMC10111261

[34] M. Gontsarik , A. Ben Mansour , L. Hong , M. Guizar‐Sicairos , S. Salentinig , J. Colloid Interface Sci. 2021, 603, 398. 34197988 10.1016/j.jcis.2021.06.050

[35] A. L. Bailey , P. R. Cullis , Biochemistry 1994, 33, 12573. 7918482 10.1021/bi00208a007

[36] H. Yu , A. Angelova , B. Angelov , B. Dyett , L. Matthews , Y. Zhang , M. El Mohamad , X. Cai , S. Valimehr , C. J. Drummond , J. Zhai , Angew. Chem. 2023, 135, 202304977. 10.1002/anie.202304977 37391876

[37] A. I. I. Tyler , H. M. G. Barriga , E. S. Parsons , N. L. C. McCarthy , O. Ces , R. V. Law , J. M. Seddon , N. J. Brooks , Soft Matter 2015, 11, 3279. 25790335 10.1039/c5sm00311c

[38] H. M. G. Barriga , M. N. Holme , M. M. Stevens , Angew. Chem., Int. Ed. 2019, 58, 2958. 10.1002/anie.201804067 PMC6606436 29926520

[39] R. Tenchov , J. M. Sasso , Q. A. Zhou , Bioconjug. Chem. 2023, 34, 941. 37162501 10.1021/acs.bioconjchem.3c00174 PMC10190134

[40] Y. Yan , X.‐Y. Liu , A. Lu , X.‐Y. Wang , L.‐X. Jiang , J.‐C. Wang , J. Controlled Release 2022, 342, 241. 10.1016/j.jconrel.2022.01.008 PMC8743282 35016918

[41] N. M. Belliveau , J. Huft , P. J. Lin , S. Chen , A. K. Leung , T. J. Leaver , A. W. Wild , J. B. Lee , R. J. Taylor , Y. K. Tam , C. L. Hansen , P. R. Cullis , Mol. Ther. Nucleic. Acids 2012, 1, e37. 23344179 10.1038/mtna.2012.28 PMC3442367

[42] S. Chen , Y. Y. C. Tam , P. J. C. Lin , M. M. H. Sung , Y. K. Tam , P. R. Cullis , J. Control. Rel. 2016, 235, 236. 10.1016/j.jconrel.2016.05.059 27238441

[43] J. A. Kulkarni , D. Witzigmann , J. Leung , Y. Y. C. Tam , P. R. Cullis , Nanoscale 2019, 11, 21733. 31713568 10.1039/c9nr09347h

[44] A. Sarode , Y. Fan , A. E. Byrnes , M. Hammel , G. L. Hura , Y. Fu , P. Kou , C. Hu , F. I. Hinz , J. Roberts , S. G. Koenig , K. Nagapudi , C. C. Hoogenraad , T. Chen , D. Leung , C.‐W. Yen , Nanoscale Adv. 2022, 4, 2107. 36133441 10.1039/d1na00712b PMC9417559

[45] P. Patel , N. M. Ibrahim , K. Cheng , Trends Pharmacol. Sci. 2021, 42, 448. 33875229 10.1016/j.tips.2021.03.002 PMC8148296

[46] R. L. Ball , K. A. Hajj , J. Vizelman , P. Bajaj , K. A. Whitehead , Nano Lett. 2018, 18, 3814. 29694050 10.1021/acs.nanolett.8b01101

[47] H. H. Ly , S. Daniel , S. K. V. Soriano , Z. Kis , A. K. Blakney , Mol. Pharm. 2022, 19, 1892. 35604765 10.1021/acs.molpharmaceut.2c00032 PMC9176215

[48] M. J. Carrasco , S. Alishetty , M.‐G. Alameh , H. Said , L. Wright , M. Paige , O. Soliman , D. Weissman , T. E. Cleveland , A. Grishaev , M. D. Buschmann , Commun. Biol. 2021, 4, 956. 34381159 10.1038/s42003-021-02441-2 PMC8358000

[49] K. A. Whitehead , J. Matthews , P. H. Chang , F. Niroui , J. R. Dorkin , M. Severgnini , D. G. Anderson , ACS Nano 2012, 6, 6922. 22770391 10.1021/nn301922x PMC3429655

[50] T. Sych , J. Schlegel , H. M. G. Barriga , M. Ojansivu , L. Hanke , F. Weber , R. Beklem Bostancioglu , K. Ezzat , H. Stangl , B. Plochberger , J. Laurencikiene , S. El Andaloussi , D. Fürth , M. M. Stevens , E. Sezgin , Nat. Biotechnol. 2024, 42, 587. 37308687 10.1038/s41587-023-01825-5 PMC11021190

[51] V. Kumar , J. Qin , Y. Jiang , R. G. Duncan , B. Brigham , S. Fishman , J. K. Nair , A. Akinc , S. A. Barros , P. V Kasperkovitz , Mol. Ther. Nucleic. Acids 2014, 3, e210. 25405467 10.1038/mtna.2014.61 PMC4459547

[52] J. Barauskas , C. Cervin , M. Jankunec , M. Špandyreva , K. Ribokaitė , F. Tiberg , M. Johnsson , Int. J. Pharm. 2010, 391, 284. 20214966 10.1016/j.ijpharm.2010.03.016

[53] M. Johnsson , N. Bergstrand , K. Edwards , J. J. R. Stålgren , Langmuir 2001, 17, 3902.

[54] L. Digiacomo , F. Cardarelli , D. Pozzi , S. Palchetti , M. A. Digman , E. Gratton , A. L. Capriotti , M. Mahmoudi , G. Caracciolo , Nanoscale 2017, 9, 17254. 29115333 10.1039/c7nr06437c PMC5700750

[55] P. J. C. Lin , Y. Y. C. Tam , I. Hafez , A. Sandhu , S. Chen , M. A. Ciufolini , I. R. Nabi , P. R. Cullis , Nanomedicine 2013, 9, 233. 22698807 10.1016/j.nano.2012.05.019

[56] O. V. Sergeeva , E. Y. Shcherbinina , N. Shomron , T. S. Zatsepin , Nucleic Acid Ther. 2022, 32, 123. 35166605 10.1089/nat.2021.0067

[57] J. Kim , S. Woo , C. M. de Gusmao , B. Zhao , D. H. Chin , R. L. DiDonato , M. A. Nguyen , T. Nakayama , C. A. Hu , A. Soucy , A. Kuniholm , J. K. Thornton , O. Riccardi , D. A. Friedman , C. M. El Achkar , Z. Dash , L. Cornelissen , C. Donado , K. N. W. Faour , L. W. Bush , V. Suslovitch , C. Lentucci , P. J. Park , E. A. Lee , A. Patterson , A. A. Philippakis , B. Margus , C. B. Berde , T. W. Yu , Nature 2023, 619, 828. 37438524 10.1038/s41586-023-06277-0 PMC10371869

[58] H. Yu , J. Iscaro , B. Dyett , Y. Zhang , S. Seibt , N. Martinez , J. White , C. J. Drummond , S. Bozinovski , J. Zhai , J. Am. Chem. Soc. 2023, 145, 24765. 10.1021/jacs.3c08729 37870621

[59] S. Palchetti , L. Digiacomo , D. Pozzi , G. Peruzzi , E. Micarelli , M. Mahmoudi , G. Caracciolo , Nanoscale 2016, 8, 12755. 27279572 10.1039/c6nr03898k

[60] H. M. G. Barriga , I. J. Pence , M. N. Holme , J. J. Doutch , J. Penders , V. Nele , M. R. Thomas , M. Carroni , M. M. Stevens , Adv. Mater. 2022, 34, 2200839. 10.1002/adma.202200839 PMC7615489 35358374

[61] C. Wang , T. Zhao , Y. Li , G. Huang , M. A. White , J. Gao , Adv. Drug Deliv. Rev. 2017, 113, 87. 27612550 10.1016/j.addr.2016.08.014 PMC5339051

[62] P. W. Schmidt , J. Appl. Crystallogr. 1991, 24, 414.

[63] J. Teixeira , J. Appl. Crystallogr. 1988, 21, 781.

[64] S. A. Dilliard , Q. Cheng , D. J. Siegwart , Engineering 2021, 118, 2109256118. 10.1073/pnas.2109256118 PMC8719871 34933999

[65] A. Gallud , M. J. Munson , K. Liu , A. Idström , H. M. G Barriga , S. R. Tabaei , N. Aliakbarinodehi , M. Ojansivu , Q. Lubart , J. J. Doutch , M. N. Holme , L. Evenäs , L. Lindfors , M. M. Stevens , A. Collén , A. Sabirsh , F. Höök , A. E. K Esbjörner , 2021, 10.1101/2021.08.20.457104.

[66] L. Li , R. Wang , D. Wilcox , A. Sarthy , X. Lin , X. Huang , L. Tian , P. Dande , R. D. Hubbard , T. M. Hansen , C. Wada , X. Zhao , W. M. Kohlbrenner , S. W. Fesik , Y. Shen , Mol. Cancer Ther. 2013, 12, 2308. 23943805 10.1158/1535-7163.MCT-12-0983-T

[67] M. J. Rust , M. Bates , X. Zhuang , Nat. Methods 2006, 3, 793. 16896339 10.1038/nmeth929 PMC2700296

[68] L. von Chamier , R. F. Laine , J. Jukkala , C. Spahn , D. Krentzel , E. Nehme , M. Lerche , S. Hernández‐Pérez , P. K. Mattila , E. Karinou , S. Holden , A. C. Solak , A. Krull , T.‐O. Buchholz , M. L. Jones , L. A. Royer , C. Leterrier , Y. Shechtman , F. Jug , M. Heilemann , G. Jacquemet , R. Henriques , Nat. Commun. 2021, 12, 2276. 33859193 10.1038/s41467-021-22518-0 PMC8050272

[69] E. Gómez‐de‐Mariscal , C. García‐López‐de‐Haro , W. Ouyang , L. Donati , E. Lundberg , M. Unser , A. Muñoz‐Barrutia , D. Sage , Nat. Methods 2021, 18, 1192. 34594030 10.1038/s41592-021-01262-9

[70] U. Schmidt , M. Weigert , C. Broaddus , G. Myers , Medical Image Computing and Computer Assisted Intervention – MICCAI 2018 2018, pp. 265–273.

[71] L. M. P. Vermeulen , T. Brans , S. K. Samal , P. Dubruel , J. Demeester , S. C. De Smedt , K. Remaut , K. Braeckmans , ACS Nano 2018, 12, 2332. 29505236 10.1021/acsnano.7b07583

[72] J. Gilleron , W. Querbes , A. Zeigerer , A. Borodovsky , G. Marsico , U. Schubert , K. Manygoats , S. Seifert , C. Andree , M. Stöter , H. Epstein‐Barash , L. Zhang , V. Koteliansky , K. Fitzgerald , E. Fava , M. Bickle , Y. Kalaidzidis , A. Akinc , M. Maier , M. Zerial , Nat. Biotechnol. 2013, 31, 638. 23792630 10.1038/nbt.2612

[73] J. P. Bost , M. Ojansivu , M. J. Munson , E. Wesén , A. Gallud , D. Gupta , O. Gustafsson , O. Saher , J. Rädler , S. G. Higgins , T. Lehto , M. N. Holme , A. Dahlén , O. Engkvist , P.‐E. Strömstedt , S. Andersson , C. I. Edvard Smith , M. M. Stevens , E. K. Esbjörner , A. Collén , S. El Andaloussi , Commun. Biol. 2022, 5, 185. 35233031 10.1038/s42003-022-03132-2 PMC8888659

[74] L. M. P. Vermeulen , S. C. De Smedt , K. Remaut , K. Braeckmans , Eur. J. Pharm. Biopharm. 2018, 129, 184. 29859281 10.1016/j.ejpb.2018.05.034

[75] A. Spadea , M. Jackman , L. Cui , S. Pereira , M. J. Lawrence , R. A. Campbell , M. Ashford , ACS Appl. Mater. Interfaces 2022, 14, 30371. 35758331 10.1021/acsami.2c06065 PMC9264317

[76] J. M. Seddon , A. M. Squires , C. E. Conn , O. Ces , A. J. Heron , X. Mulet , G. C. Shearman , R. H. Templer , Philos. Trans. R. Soc., A 2006, 364, 2635. 10.1098/rsta.2006.1844 16973480

[77] A. J. Smith , S. G. Alcock , L. S. Davidson , J. H. Emmins , J. C. Hiller Bardsley , P. Holloway , M. Malfois , A. R. Marshall , C. L. Pizzey , S. E. Rogers , O. Shebanova , T. Snow , J. P. Sutter , E. P. Williams , N. J. Terrill , J. Synchrotron Radiat. 2021, 28, 939. 33950002 10.1107/S1600577521002113 PMC8127364

[78] D. M. Owen , Methods in Membrane Lipids, Springer, New York, New York, NY 2015, 199.

[79] O. Arnold , J. C. Bilheux , J. M. Borreguero , A. Buts , S. I. Campbell , L. Chapon , M. Doucet , N. Draper , R. F Leal , M. A. Gigg , V. E. Lynch , A. Markvardsen , D. J. Mikkelson , R. L. Mikkelson , R. Miller , K. Palmen , P. Parker , G. Passos , T. G. Perring , P. F. Peterson , S. Ren , M. A. Reuter , A. T. Savici , J. W. Taylor , R. J. Taylor , R. Tolchenov , W. Zhou , J. Zikovsky , Nucl. Instrum. Methods Phys. Res. A 2014, 764, 156.

[80] S.‐H. Kang , M.‐J. Cho , R. Kole , Biochemistry 1998, 37, 6235. 9572837 10.1021/bi980300h

[81] A. D. Edelstein , M. A. Tsuchida , N. Amodaj , H. Pinkard , R. D. Vale , N. Stuurman , J. Biol. Methods 2014, 1, 1. 10.14440/jbm.2014.36 PMC4297649 25606571

[82] M. Ovesný , P. Křížek , J. Borkovec , Z. Švindrych , G. M. Hagen , Bioinformatics 2014, 30, 2389. 24771516 10.1093/bioinformatics/btu202 PMC4207427

[83] J. Schindelin , I. Arganda‐Carreras , E. Frise , V. Kaynig , M. Longair , T. Pietzsch , S. Preibisch , C. Rueden , S. Saalfeld , B. Schmid , J.‐Y. Tinevez , D. J. White , V. Hartenstein , K. Eliceiri , P. Tomancak , A. Cardona , Nat. Methods 2012, 9, 676. 22743772 10.1038/nmeth.2019 PMC3855844

[84] C. McQuin , A. Goodman , V. Chernyshev , L. Kamentsky , B. A. Cimini , K. W. Karhohs , M. Doan , L. Ding , S. M. Rafelski , D. Thirstrup , W. Wiegraebe , S. Singh , T. Becker , J. C. Caicedo , A. E. Carpenter , PLoS Biol. 2018, 16, 2005970. 10.1371/journal.pbio.2005970 PMC6029841 29969450

[85] D. R. Stirling , M. J. Swain‐Bowden , A. M. Lucas , A. E. Carpenter , B. A. Cimini , A. Goodman , BMC Bioinformatics 2021, 22, 433. 34507520 10.1186/s12859-021-04344-9 PMC8431850

