# Supplementary material for: Formulation and Characterization of Novel Ionizable and Cationic Lipid Nanoparticles for the Delivery of Splice‐Switching Oligonucleotides
Source: Adv Mater. 2025 Mar 16;37(17):2419538. doi: 10.1002/adma.202419538 (PMC12038542; doi:10.1002/adma.202419538)
Supplement: Supplementary file 1 — Supporting Information [file ADMA-37-2419538-s001.docx]

**Supporting information**

**Formulation and Characterization of Novel Ionizable and Cationic Lipid Nanoparticles for the Delivery of Splice-Switching Oligonucleotides**

*Miina Ojansivu^1^, Hanna M. G. Barriga^1*^, Margaret N. Holme^1^, Stefanie Morf^1^, James J. Doutch^2^, Samir EL Andaloussi^3,4,5^, Tomas Kjellman^6^, Markus Johnsson^6^, Justas Barauskas^6^, Molly M. Stevens^1,7*^*

1 Department of Medical Biochemistry and Biophysics, Karolinska Institute, Stockholm, 171 77, Sweden

2 ISIS Muon and Neutron Source, Rutherford Appleton Laboratory, Harwell Campus, Oxfordshire OX11 0QX, United Kingdom

3 Division of Biomolecular and Cellular Medicine, Department of Laboratory Medicine, Karolinska Institute, 14152 Huddinge, Stockholm, Sweden

4 Department of Cellular Therapy and Allogeneic Stem Cell Transplantation (CAST), Karolinska University Hospital, 141 86 Stockholm, Sweden

5 Karolinska ATMP Center, Karolinska Institute, 14152 Huddinge, Stockholm, Sweden

6 Camurus AB**,** Ideon Science Park, Lund, 223 70, Sweden

7 Kavli Institute for Nanoscience Discovery, Department of Physiology, Anatomy and Genetics, and Department of Engineering Science, University of Oxford, OX1 3QU Oxford, UK

*Corresponding authors:

Molly M. Stevens

Department of Physiology, Anatomy, and Genetics

Kavli Institute for Nanoscience Discovery

University of Oxford

South Parks Rd, Oxford, OX1 3QU

UK

Tel: +44 (0)20 7594 6804

Email: molly.stevens@dpag.ox.ac.uk

Hanna Barriga

Department of Medical Biochemistry and Biophysics

Karolinska Institute

171 77 Stockholm

Sweden

Tel: +46 72 250 99 37

Email: [hanna.barriga@ki.se](mailto:hanna.barriga@ki.se)

**STORM image reconstruction parameters used in ThunderSTORM**

Image Filtering

Filter: Difference-of-Gaussians filter (Sigma1 = 1.0 px, Sigma2 = 1.6 px)

Approximate localization of molecules

Method: Local maximum

Peak intensity threshold: std(Wave.F1)

Connectivity: 8-neighbourhood

Sub-pixel localization of molecules

Method: PSF: Integrated Gaussian

Fitting radius (px): 3

Fitting method: Weighted least squares

Initial sigma (px): 1.6

Multi-emitter fitting analysis: enabled

Maximum of molecules per fitting region: 3

Model selection threshold (p-value): 1.0E-6

The intensity range (photons) was not limited.

The lower and upper limits for sigma filtering were calculated using the full width at half maximum (FWHM) formula for resolution (FWHM = λ/2NA, where λ is the wavelength (here 642 nm) and NA=1.49). This gave the upper limit for the sigma filtering. The lower limit was set to one standard deviation of the fitted Gaussian curve (FWHM/2.3). Thus, the upper and lower sigma filtering values for the 642 nm channel were [90, 215]. For the intensity-based filtering a fixed lower limit of 1500 was chosen based on visual evaluation and used for all the images.


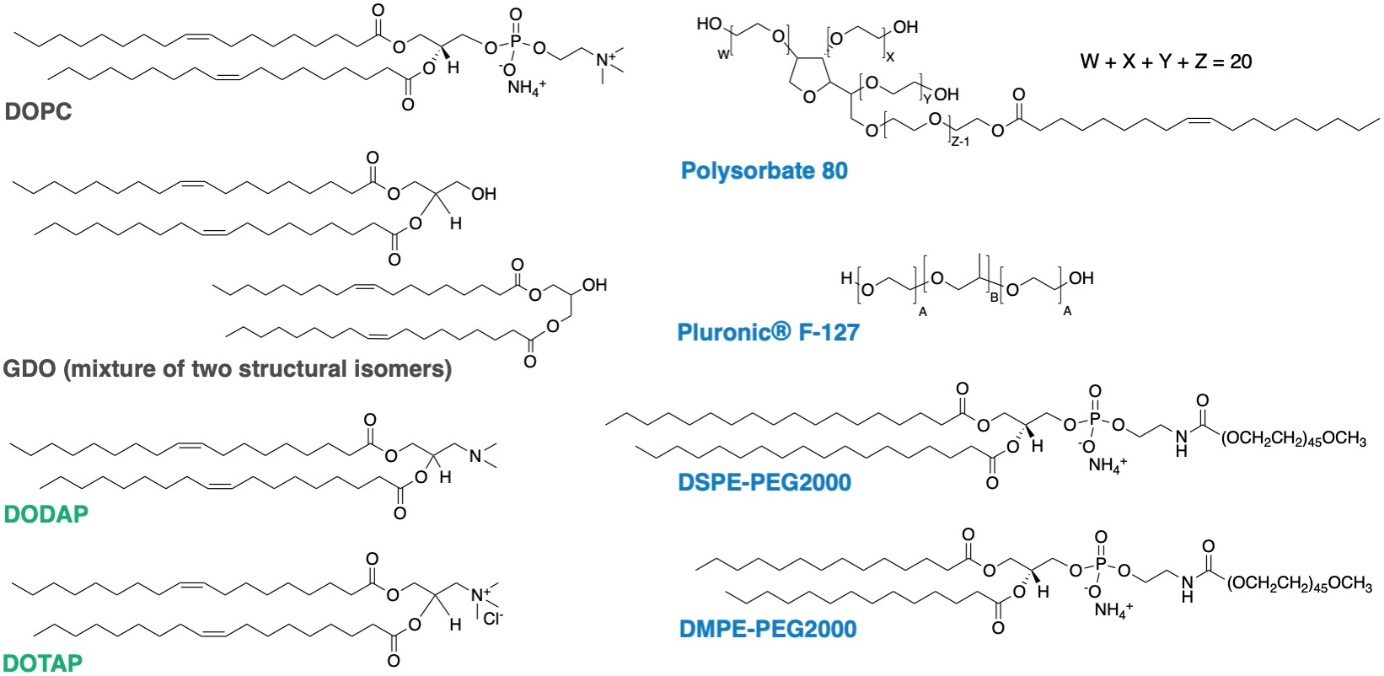


**Figure S1.** Chemical structures of the used lipids and stabilizers.


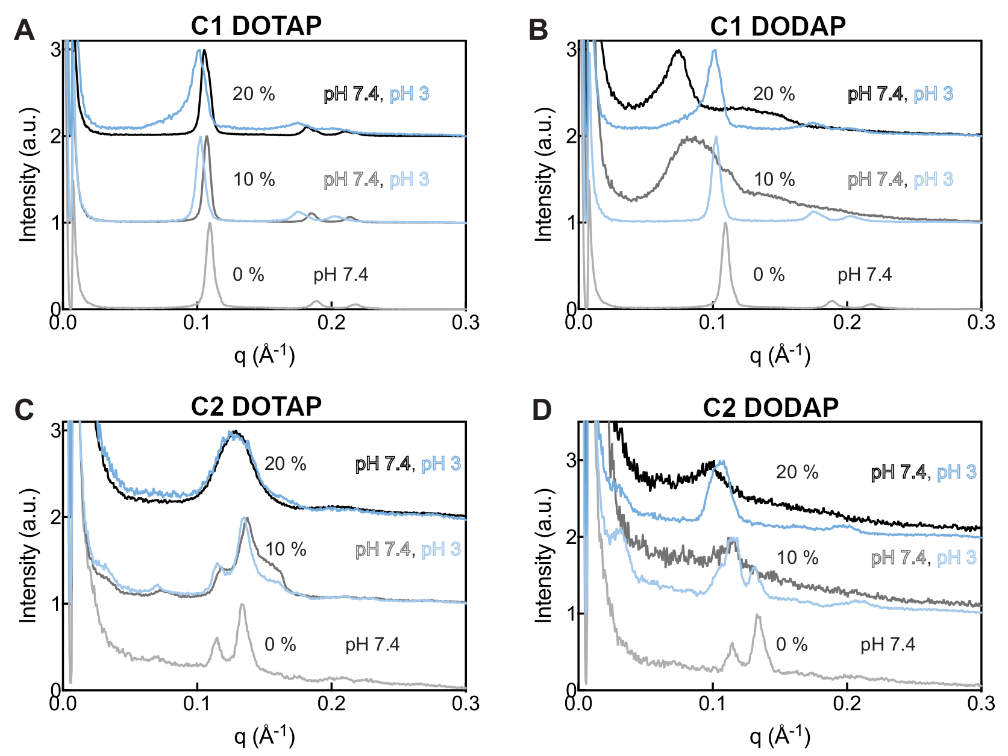


**Figure S2.** Bulk SAXS data (N = 1) of lipid compositions C1DOTAP, C1DODAP, C2DOTAP, C2DODAP measured at 25 °C in citrate buffer either pH3 or pH 7.4 with either 0, 10 or 20 mol-% DOTAP / DODAP. Note that for 0 mol-% the DODAP / DOTAP samples are the same sample but plotted in both (A,B) or (C,D) for data clarity. Samples have been offset on the y axis for clarity.

**
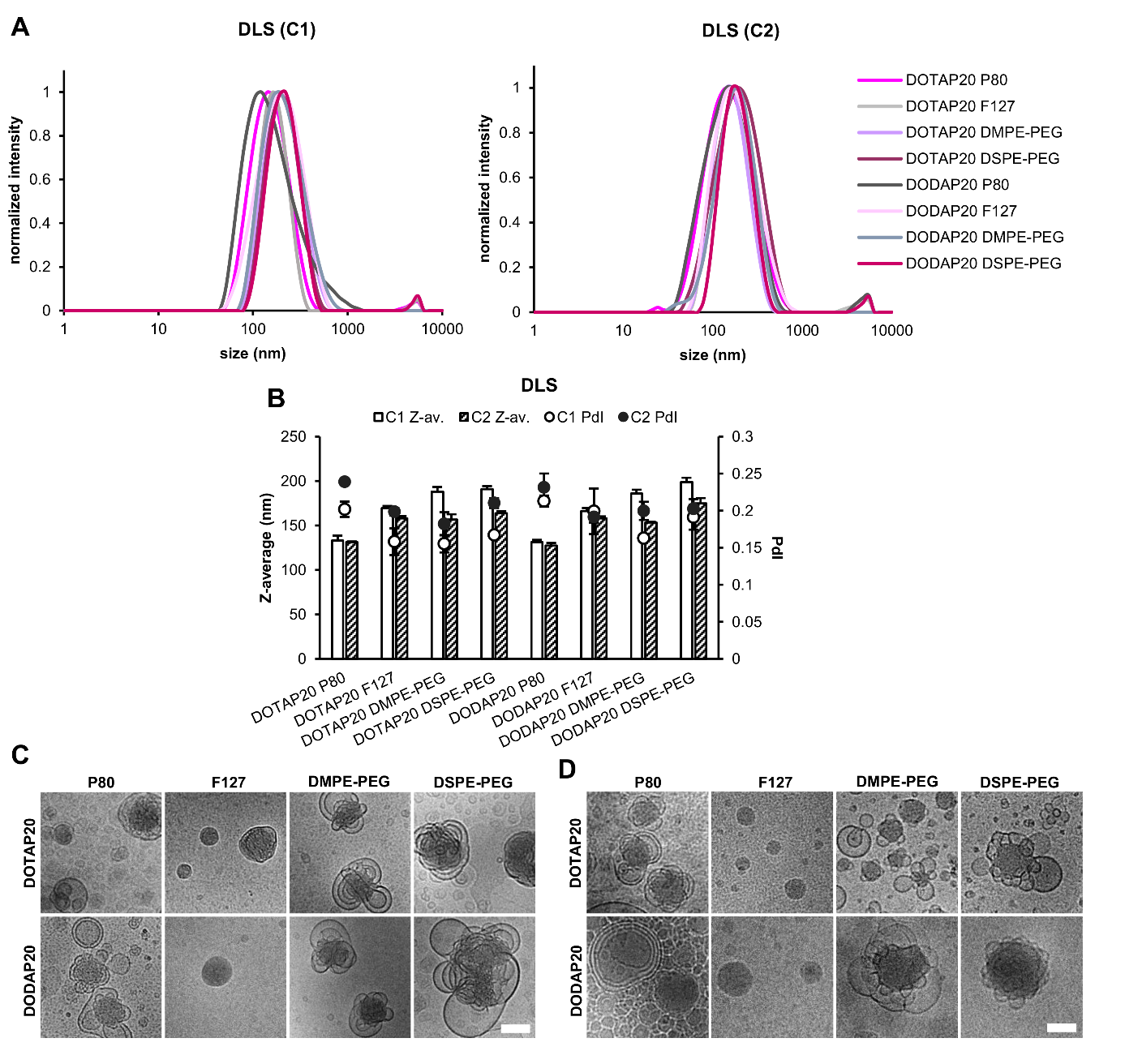
**

**Figure S3.** LNP size and morphology in pH 3 citrate buffer. **A.** Representative DLS intensity plots for the 16 novel LNPs, measured in pH 3 citrate buffer. **B.** Average LNP hydrodynamic diameter (Z-average) and polydispersity (polydispersity index, PdI) in pH 3 citrate buffer, analyzed by DLS. N = 3. Error bars represent standard deviation. **C,D.** Representative cryoEM visualizations of the morphology of novel LNP series C1 and C2, respectively, in pH 3 citrate buffer. Scale bars 100 nm.

**TNS assay**

For the 6-(p-toluidino)-2-naphthalenesulfonyl chloride (TNS) assay, 20 mM citrate/Na-citrate buffer (with 150 mM NaCl, pH 3.0-6.6), 20 mM Tris-HCl buffer (with 150 mM NaCl, pH 7.1-8.9), and 20 mM Na-carbonate/Na-bicarbonate buffer (with 150 mM NaCl pH 9.5-10.8) were prepared. 10 mM TNS stock in DMSO was further dissolved in water to 120 µM assay reagent. Lipid nanoparticles were dialyzed against PBS to reach neutral pH for the TNS assay and the dialyzed LNPs were further diluted to 500 µM with PBS. In 96-well plate 5 µL of the 500 µM LNPs and 5 µL of the TNS assay reagent were mixed with 90 µL of buffer. Followed by 10 min incubation at room temperature the fluorescence was read at excitation of 320 nm and emission of 450 nm. All the samples were measured in duplicates. The pKa values of the DODAP and MC3 control LNPs were determined as the pH at which the LNP sample showed 50 % of the maximum fluorescence. All the DOTAP LNPs had high fluorescent values across the whole pH range studied, indicative of their permanent positive charge. Thus, the pKa value determination for these samples was concluded as not being feasible.


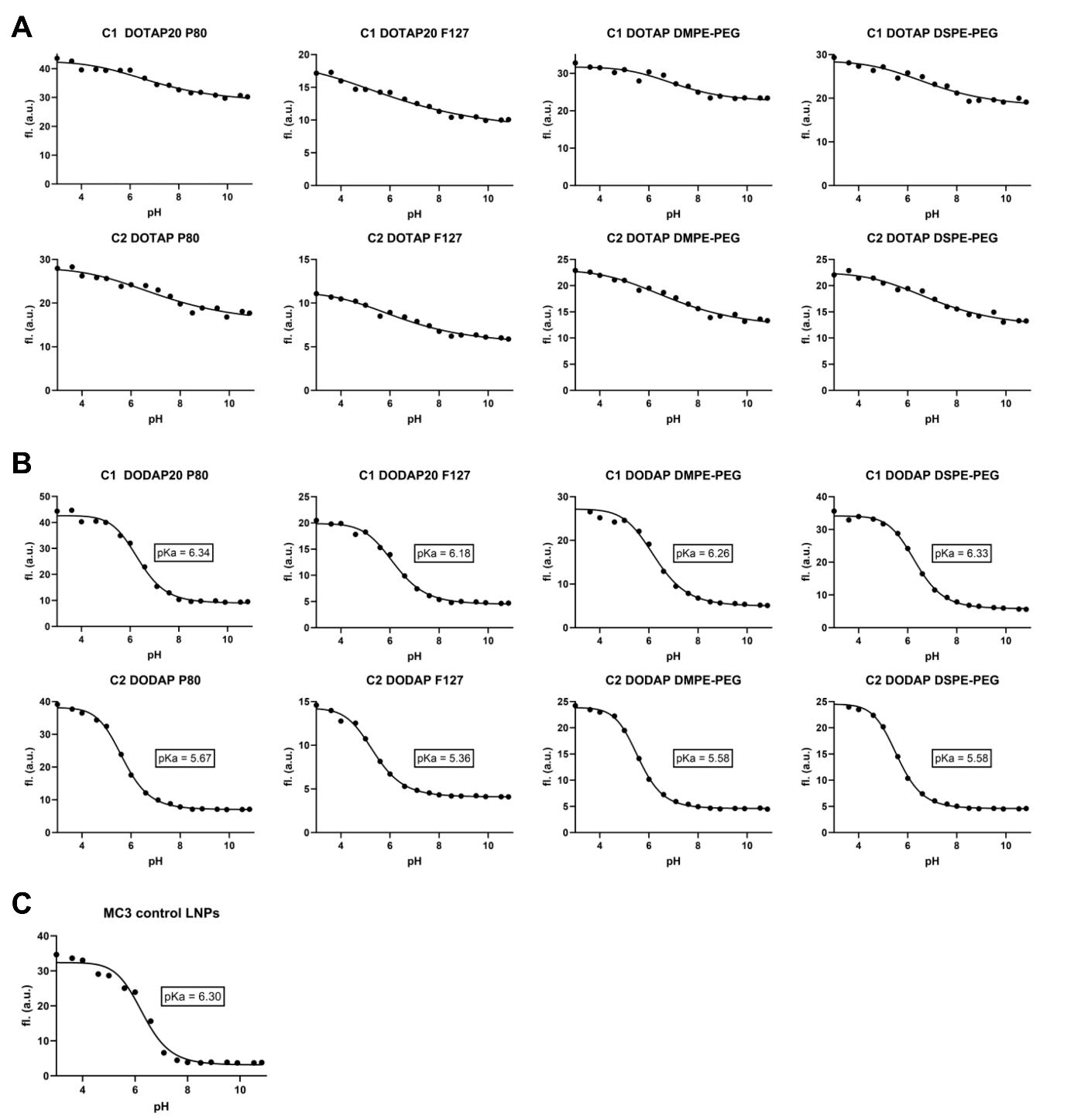


**Figure S4.** LNP pKa determination using a TNS assay. **A.** DOTAP LNPs. **B.** DODAP LNPs. **C.** MC3 control LNPs. N = 1, n = 2.

**
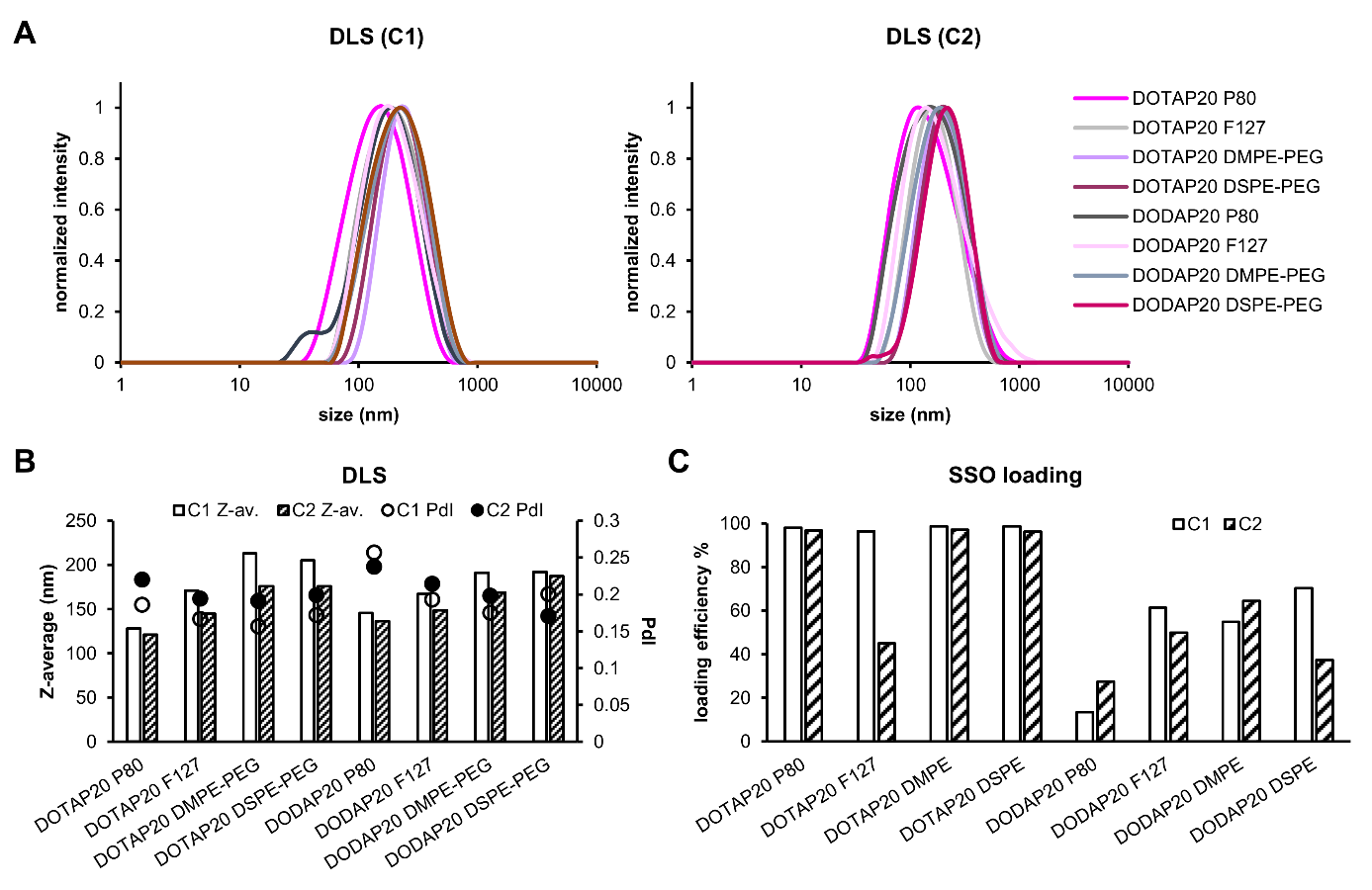
**

**Figure S5.** LNP size and SSO loading efficiency after dialysis against PBS (pH 7). **A.** Representative DLS intensity plots for the 16 novel LNPs, measured in PBS. **B.** Average LNP hydrodynamic diameter (Z-average) and polydispersity (polydispersity index, PdI) in PBS, analyzed by DLS. N = 1. **C.** SSO cargo loading efficiency % into the novel LNPs, analyzed by RiboGreen assay. N = 1.


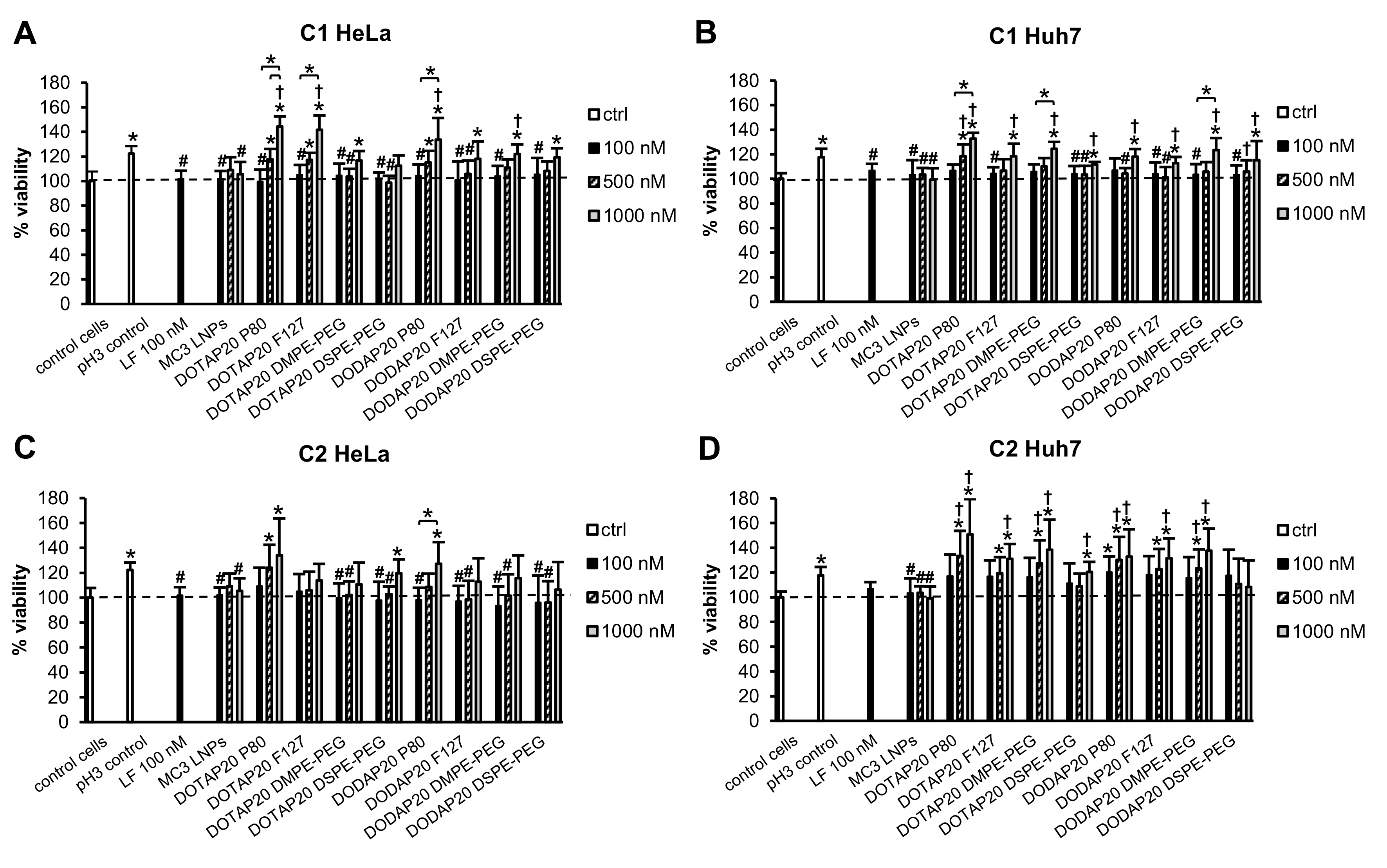


**Figure S6.** Cell viability after 4 h LNP treatment. **A, B.** HeLa and Huh7 cells treated with C1 LNPs, respectively. **C, D.** HeLa and Huh7 cells treated with C2 LNPs, respectively. Cell viability was evaluated with alamarBlue assay. N = 3, n = 9 for the LNP samples and n = 18 for the control cells, pH 3 control and Lipofectamine (LF) control. * < 0.05 (compared to the control cells unless otherwise indicated), # < 0.05 (compared to the pH3 control). † < 0.05 (compared to the MC3 LNPs). Kruskal-Wallis test with Dunn’s post hoc correction. Data is shown as means plus standard deviation.

**Hemolysis assay**

Hemolysis assay for the LNPs was performed as described by Peeler et al. ^[1]^. Briefly, red blood cells (RBCs) were isolated from fresh human blood obtained from Blodcentralen Odenplan, Karolinska University Hospital (ethical permit 2023-06187-01, Swedish Ethical Review Authority (Etikprövningsmyndigheten), Modifiering av immunceller), by centrifugation. After plasma removal the RBCs were washed three times with 150 mM NaCl and finally suspended in pH 7.4 phosphate buffer (100 mM). The assay was conducted on a 96-well plate where the RBCs were incubated with various SSO concentrations (0-33 μg/mL) for 1 h at + 37 °C. Following the incubation the plate was centrifuged and the supernatants (containing the released hemoglobin) were transferred to a new 96-well plate and the absorbance was measured at 541 nm with Varioskan LUX Multimode Microplate Reader (Thermo Fisher Scientific). Hemolysis percentage was calculated relative to the 1 % Triton X-100 positive control.


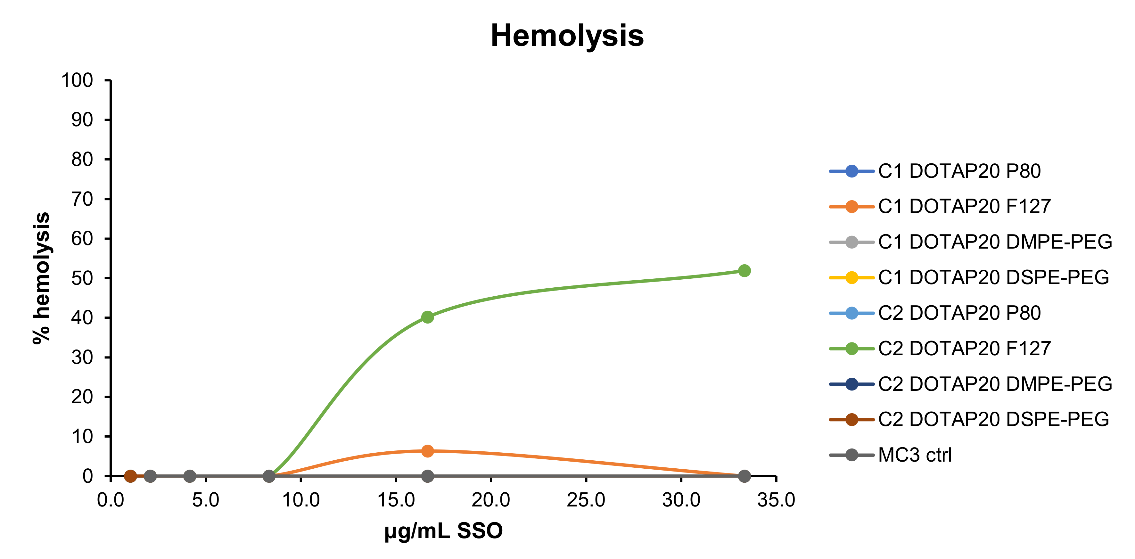


**Figure S7.** Evaluation of LNP-induced hemolysis. Hemolysis assay was conducted at pH 7.4 using freshly isolated human erythrocytes. N=1.


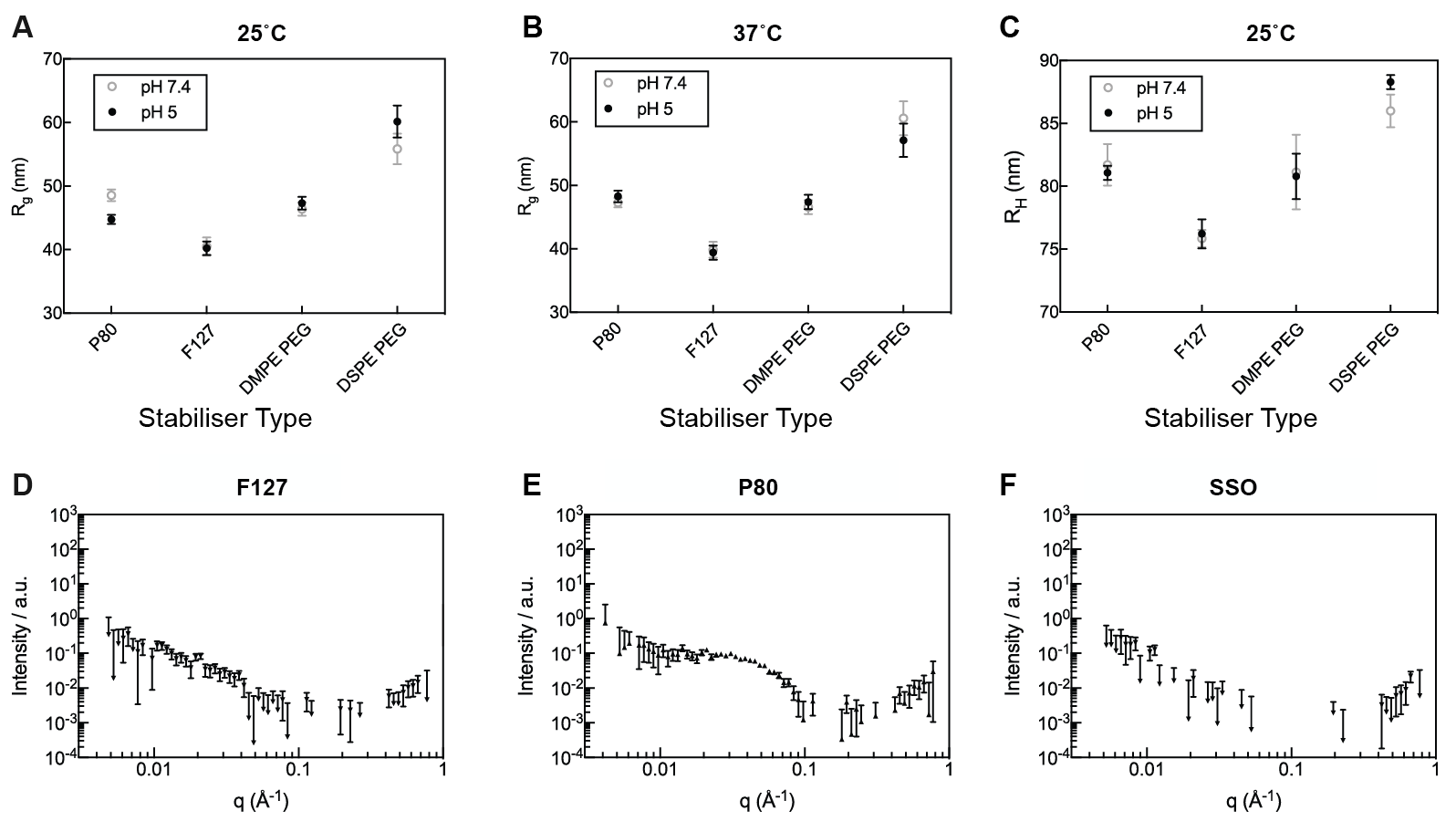


**Figure S8.** LNP data extracted from SANS fitting and DLS. **A,B.** Fitting of the data using the Unified Model fit to extract the radius of gyration (R_g_) where the fit data was collected at 25, 37°C with samples diluted in either pH5, pH 7.4 citrate buffer. Error bars represent the error in the fitting (N = 1) **C.** DLS data collected from LNP samples at 25°C where the R_H_ is represented by the Z average intensity mean and the error bars represent the standard deviation in a triplicate measurement (N = 1, n = 3). **D,E,F** SANS data collected for F127, P80, SSO in solution at concentrations representing the maximum solution concentration if none of the components are integrated into the LNP (i.e. the maximum possible concentration).


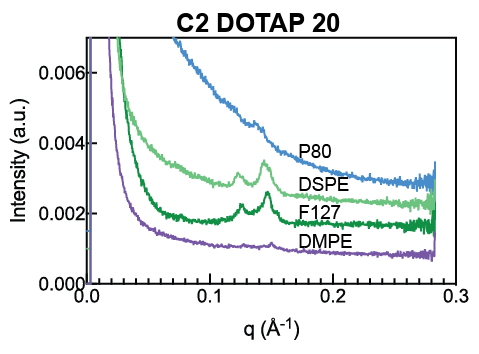


**Figure S9.** Bulk SAXS data (N = 1) of lipid composition C2 DOTAP20 with stabiliser at specific mol-% (see Table 1) homogenously distributed throughout the sample (via freeze thaw), measured at 25°C in pH 7.4 citrate buffer.

**
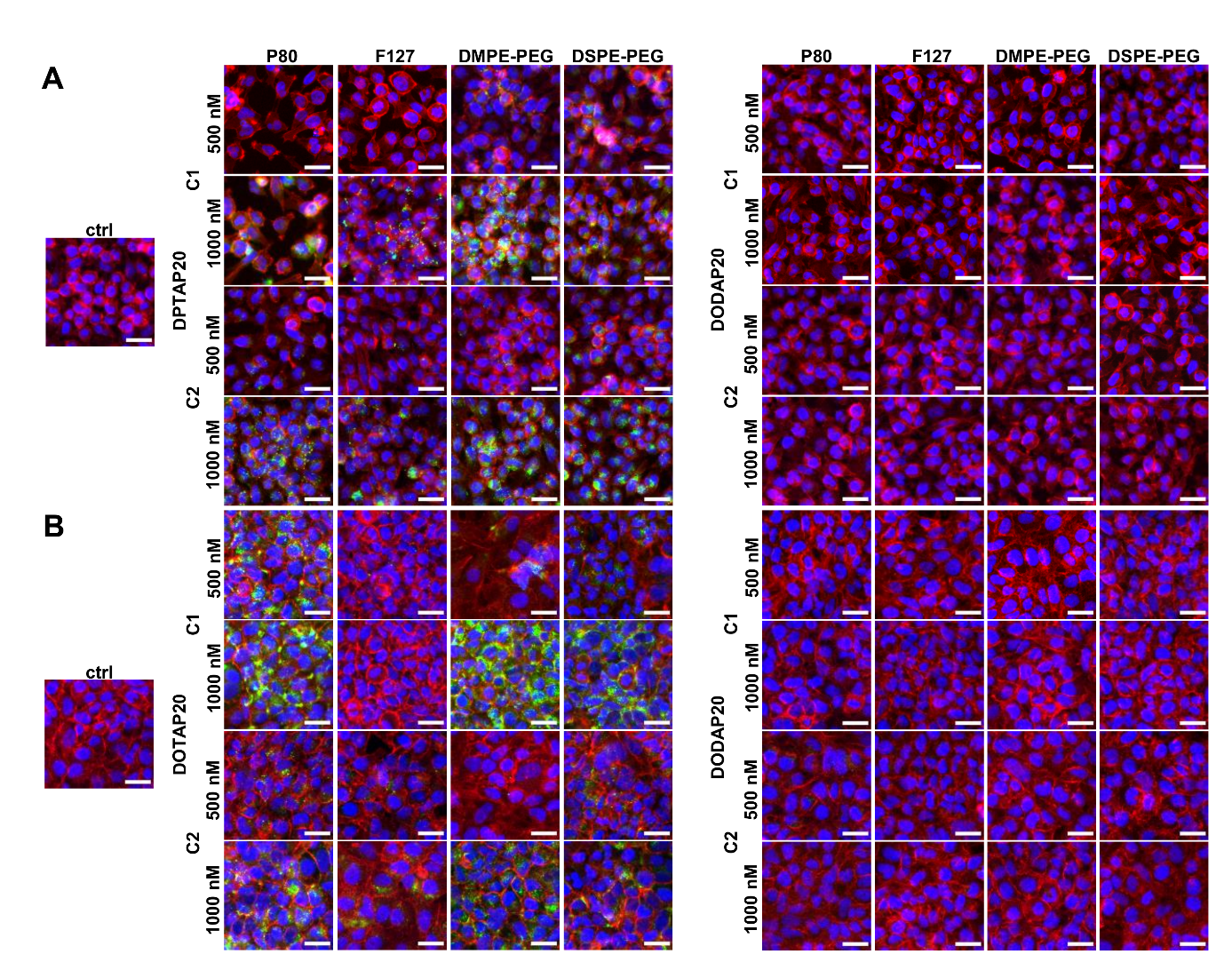
**

**Figure S10.** LNP cell uptake at 4 h. **A, B.** Representative images of LNP uptake into HeLa and Huh7 cells, respectively. DOTAP20 LNPs on the left side and DODAP20 on the right side. DOTAP20 LNPs on the left side and DODAP20 on the right side. B. Huh7 cells. DOTAP20 LNPs on the left side and DODAP20 on the right side. Rhodamine B-labelled LNPs = green, actin cytoskeleton labelled with phalloidin Alexa Fluor 647 = red, nuclei labelled with DAPI = blue. Scale bars 30 μm.


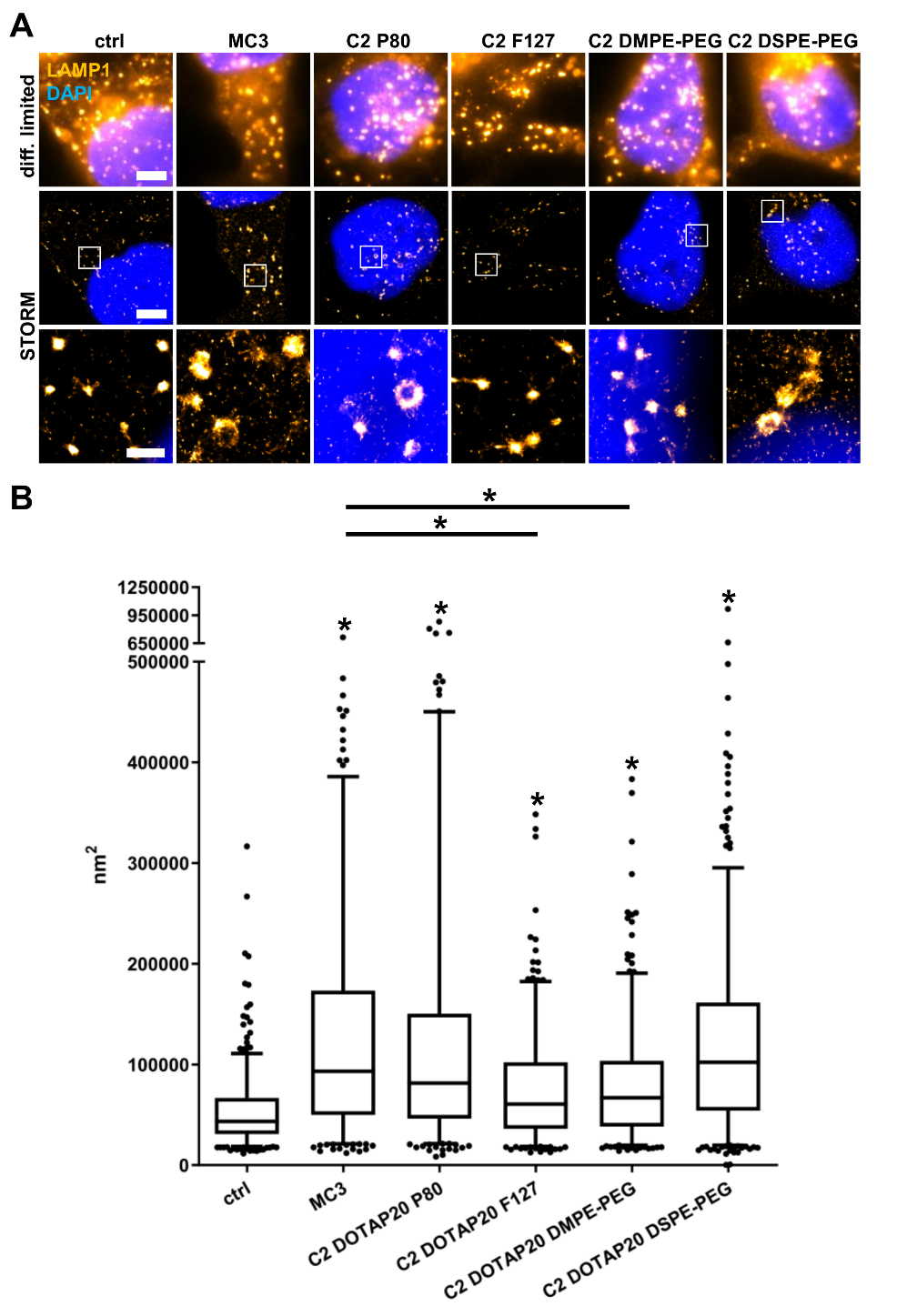


**Figure S11.** STORM imaging of lysosomes after 4 h cell treatment with LNPs. **A.** Representative images of HeLa lysosomes. Anti-LAMP1 staining (yellow), nuclei stained with DAPI (blue). Scale bars: diffraction-limited and full view STORM 5 μm, zoom-in STORM 1 μm. **B.** Quantification of lysosome sizes in HeLa cells at 4 h with a deep learning method. *p < 0.05 compared to the ctrl unless otherwise indicated. Kruskal-Wallis test with Dunn’s post hoc correction. The box extends from the 25^th^ to 75^th^ percentile, the line in the middle of the box represents median and the whiskers show 5 and 95 percentiles. The number of images and identified objects (lysosomes) in the quantification are indicated in **Table S5**.

**Table S1.** Number of images and identified objects (lysosomes) in the quantification of STORM 24 h HeLa images.

|  | **ctrl** | **MC3** | **C1 DOTAP20 P80** | **C1 DOTAP20 F127** | **C1 DOTAP20 DMPE-PEG** | **C1 DOTAP20 DSPE-PEG** | **C2 DOTAP20 P80** | **C2 DOTAP20 F127** | **C2 DOTAP20 DMPE-PEG** | **C2 DOTAP20 DSPE-PEG** |
| --- | --- | --- | --- | --- | --- | --- | --- | --- | --- | --- |
| **n images** | 7 | 7 | 7 | 7 | 7 | 7 | 6 | 7 | 7 | 7 |
| **n objects** | 399 | 380 | 496 | 398 | 498 | 442 | 492 | 467 | 501 | 462 |

**Table S2.** Number of images and identified objects (lysosomes) in the quantification of STORM 24 h Huh7 images.

|  | **ctrl** | **MC3** | **C1 DOTAP20 P80** | **C1 DOTAP20 F127** | **C1 DOTAP20 DMPE-PEG** | **C1 DOTAP20 DSPE-PEG** | **C2 DOTAP20 P80** | **C2 DOTAP20 F127** | **C2 DOTAP20 DMPE-PEG** | **C2 DOTAP20 DSPE-PEG** |
| --- | --- | --- | --- | --- | --- | --- | --- | --- | --- | --- |
| **n images** | 6 | 6 | 6 | 6 | 6 | 6 | 6 | 6 | 6 | 6 |
| **n objects** | 284 | 279 | 357 | 344 | 393 | 343 | 433 | 448 | 443 | 302 |

**Table S3.** Results from Power Law fitting of SANS data.

| **Sample** |  | **pH** | **Temperature (℃)** | **Power Law** | **Error** | **Chi^2^** |
| --- | --- | --- | --- | --- | --- | --- |
| C2_DOTAP20_P80_SSO |  | pH 7.4 | 37 | 3.7558 | 0.018923 | 1.4156 |
| C2_DOTAP20_P80_SSO |  | pH 5 | 37 | 3.7651 | 0.0167 | 1.66 |
| C2_DODAP20_P80_SSO |  | pH 7.4 | 37 | 3.524 | 0.016687 | 1.4345 |
| C2_DODAP20_P80_SSO |  | pH 5 | 37 | 3.4555 | 0.016349 | 0.8802 |
| C1_DOTAP20_P80_SSO |  | pH 7.4 | 37 | 3.6387 | 0.027 | 3.0102 |
| C1_DOTAP20_P80_SSO |  | pH 5 | 37 | 3.7413 | 0.045953 | 2.6108 |
| C1_DODAP20_P80_SSO |  | pH 7.4 | 37 | 3.3625 | 0.023036 | 1.4197 |
| C1_DODAP20_P80_SSO |  | pH 5 | 37 | 3.5396 | 0.024776 | 2.2602 |

**Table S4.** Results from Unified model fitting of SANS data.

| **Sample** | **pH** | **Temperature (℃)** | **Rg1 (Å)** | **Error** | **Power 1** | **Error** | **B1 - porod** | **Error** | **G1 - guinier** | **Error** | **Chi2** | **Rg1 (nm)** | **Error** |
| --- | --- | --- | --- | --- | --- | --- | --- | --- | --- | --- | --- | --- | --- |
| C2_DOTAP20_P80_SSO | pH 7.4 | 25 | 486 | 9 | 3,53 | 0,02 | 1,5E-06 | 1,0E-07 | 1509 | 99 | 10,3 | 48,6 | 0,9 |
| C2_DOTAP20_P80_SSO | pH 5 | 25 | 448 | 7 | 3,50 | 0,02 | 1,6E-06 | 1,1E-07 | 1129 | 62 | 13,8 | 44,8 | 0,7 |
| C2_DOTAP20_F127_SSO | pH 7.4 | 25 | 405 | 14 | 4,30 | 0,06 | 2,7E-08 | 7,4E-09 | 236 | 20 | 1,0 | 40,5 | 1,4 |
| C2_DOTAP20_F127_SSO | pH 5 | 25 | 402 | 11 | 4,31 | 0,04 | 2,5E-08 | 4,3E-09 | 224 | 13 | 1,6 | 40,2 | 1,1 |
| C2_DOTAP20_DSPEPEG_SSO | pH 7.4 | 25 | 559 | 24 | 3,81 | 0,02 | 3,7E-07 | 3,1E-08 | 1323 | 155 | 1,3 | 55,9 | 2,4 |
| C2_DOTAP20_DSPEPEG_SSO | pH 5 | 25 | 602 | 25 | 3,76 | 0,02 | 4,5E-07 | 3,2E-08 | 1566 | 172 | 1,7 | 60,2 | 2,5 |
| C2_DOTAP20_DMPEPEG_SSO | pH 7.4 | 25 | 464 | 11 | 4,17 | 0,02 | 9,7E-08 | 8,8E-09 | 945 | 52 | 3,6 | 46,4 | 1,1 |
| C2_DOTAP20_DMPEPEG_SSO | pH 5 | 25 | 473 | 10 | 4,19 | 0,02 | 9,0E-08 | 7,3E-09 | 1002 | 54 | 3,2 | 47,3 | 1,0 |
| C2_DOTAP20_P80_SSO | pH 7.4 | 37 | 473 | 7 | 3,56 | 0,02 | 1,3E-06 | 9,7E-08 | 1417 | 75 | 6,6 | 47,3 | 0,7 |
| C2_DOTAP20_P80_SSO | pH 5 | 37 | 483 | 9 | 3,62 | 0,02 | 1,0E-06 | 8,2E-08 | 1558 | 100 | 11,9 | 48,3 | 0,9 |
| C2_DOTAP20_F127_SSO | pH 7.4 | 37 | 399 | 13 | 4,31 | 0,04 | 2,6E-08 | 5,2E-09 | 231 | 16 | 2,7 | 39,9 | 1,3 |
| C2_DOTAP20_F127_SSO | pH 5 | 37 | 394 | 11 | 4,35 | 0,04 | 2,1E-08 | 3,6E-09 | 223 | 15 | 1,6 | 39,4 | 1,1 |
| C2_DOTAP20_DSPEPEG_SSO | pH 7.4 | 37 | 606 | 27 | 3,77 | 0,02 | 4,3E-07 | 3,1E-08 | 1494 | 140 | 1,4 | 60,6 | 2,7 |
| C2_DOTAP20_DSPEPEG_SSO | pH 5 | 37 | 571 | 26 | 3,82 | 0,02 | 3,4E-07 | 2,9E-08 | 1311 | 159 | 1,6 | 57,1 | 2,6 |
| C2_DOTAP20_DMPEPEG_SSO | pH 7.4 | 37 | 466 | 11 | 4,22 | 0,02 | 7,8E-08 | 6,4E-09 | 935 | 55 | 2,3 | 46,6 | 1,1 |
| C2_DOTAP20_DMPEPEG_SSO | pH 5 | 37 | 474 | 11 | 4,22 | 0,02 | 7,8E-08 | 6,7E-09 | 986 | 57 | 2,6 | 47,4 | 1,1 |

**Table S5.** Number of images and identified objects (lysosomes) in the quantification of STORM 4 h images.

|  | **ctrl** | **MC3** | **C2 DOTAP20 P80** | **C2 DOTAP20 F127** | **C2 DOTAP20 DMPE-PEG** | **C1 DOTAP20 DSPE-PEG** |
| --- | --- | --- | --- | --- | --- | --- |
| **n images** | 5 | 5 | 5 | 5 | 5 | 5 |
| **n objects** | 413 | 336 | 320 | 329 | 335 | 439 |

**References**

[1] D. J. Peeler, S. N. Thai, Y. Cheng, P. J. Horner, D. L. Sellers, S. H. Pun, *Biomaterials* 2019, *192*, 235.
